# The evolutionary history of Cytochrome P450 genes in four filamentous Ascomycetes

**DOI:** 10.1186/1471-2148-7-30

**Published:** 2007-02-26

**Authors:** Jixin Deng, Ignazio Carbone, Ralph A Dean

**Affiliations:** 1 Center for Integrated Fungal Research, North Carolina State University, Raleigh, NC 27695, USA; 2 Department of Biology, University of North Carolina at Chapel Hill, Chapel Hill, NC 27599, USA

## Abstract

**Background:**

The Cytochrome P450 system is important in fungal evolution for adapting to novel ecological niches. To elucidate the evolutionary process of cytochrome P450 genes in fungi with different life styles, we studied the patterns of gene gains and losses in the genomes of four filamentous Ascomycetes, including two saprotrophs (*Aspergillus nidulans *(AN) and *Neurospora crassa *(NC)) and two plant pathogens (*Fusarium graminearum *(FG) and *Magnaporthe grisea *(MG)).

**Results:**

A total of 376 P450 genes were assigned to 168 families according to standard nomenclature. On average, only 1 to 2 genes per family were in each genome. To resolve conflicting results between different clustering analyses and standard family designation, a higher order relationship was formulated. 376 genes were clustered into 115 clans. Subsequently a novel approach based on parsimony was developed to build the evolutionary models. Based on these analyses, a core of 30 distinct clans of P450s was defined. The core clans experienced contraction in all four fungal lineages while new clans expanded in all with exception of NC. MG experienced more genes and clans gains compared to the other fungi. Parsimonious analyses unanimously supported one species topology for the four fungi.

**Conclusion:**

The four studied fungi exhibit unprecedented diversity in their P450omes in terms of coding sequence, intron-exon structures and genome locations, suggesting a complicated evolutionary history of P450s in filamentous Ascomycetes. Clan classification and a novel strategy were developed to study evolutionary history. Contraction of core clans and expansion of novel clans were identified. The exception was the NC lineage, which exhibited pure P450 gene loss.

## Background

Fungi comprise a large and diverse kingdom of organisms. It is estimated that as many as 1.5 million species exist in the planet today [1,2]. Most described fungi grow by producing long, multi-celled hyphae, and are known as filamentous fungi. Filamentous fungi occupy a wide range of ecological niches with diverse life histories and physiological processes. Many live as saprotrophs decomposing and absorbing nutrients from dead materials while others have evolved the ability to be pathogens deriving their nutrients from living or dying hosts. Taking advantage of available genome sequences to explore the evolution of important gene families may help shed light on the processes that have allowed fungi to exploit diverse habitats.

The P450-containing monooxygenase system, an ancient multicomponent electron transfer chain system, plays an important role in a myriad of hydroxylation and oxidation processes leading to degradation, detoxification and syntheses of life critical compounds. P450 proteins, as the terminal oxidases of the system, are the ideal materials for evolutionary studies of biodiversity and adaptation for several reasons. First of all, P450 enzymes form a superfamily with similar heme-thiolate structures that are distributed widely throughout life forms. This implies a very early origin of this superfamily. The first P450 gene may have emerged more than 3.5 billion years ago, shortly after the origin of terrestrial life [3]. Secondly, the P450 superfamily consists of a large number of genes, contributing a broad array of biological functions in individual organisms. In addition to housekeeping functions, many have highly specialized function as the product of adaptive evolution. For example, pisatin demethylase gene (PDA) from *Nectria *species detoxifies one specific class of the plant defence compound [4]. On the other hand, P450s in fungi are required for biosynthesis of several secondary metabolites such as toxins and hormones. Thirdly, expansion and divergence of P450s has closely paralleled the evolution and coevolution of organisms. In fact, P450 gene evolution is thought to be correlated with historical biota evolution and atmosphere oxygen concentration flux [3]. Specifically, the occurrence of certain important biota in history has been linked with a major branching of P450s followed by a great expansion of the diverged branch. In a case study of coevolution of plants and animals, it was proposed that intraspecific and interspecific polymorphisms of P450s in the predator (animals) and prey (plants) may be the product of the evolutionary warfare between them, i.e. plants are driven to produce defence chemicals-toxins while animals strive to produce new detoxifying genes [5]. For example the expansion of CYP6 gene family in the swallowtail butterfly appears to be linked to the ability to detoxify xanthotoxin [6].

In spite of the wide sequence diversity and function of P450s, certain sequence motifs corresponding to the conserved tertiary structure and enzyme functions are identifiable. The signature motif (F-G-R-C-G) is required to bind heme, however, only cysteine is absolutely conserved across all P450 genes. Another motif called the E-R-R triad is thought to be important for locking the heme pocket into position and to assure stabilization of the conserved core structure. The E-R-R triad lies in the K-helix beginning with consensus (E-R) and ending with the Arg in the "PER" consensus. A third relatively conserved motif is the I-helix oxygen binding domain [A/G]G-[E/D]T [T/S]. All these motifs are short overall and somewhat variable. To group members within the P450 superfamily, genes are assigned into families and subfamilies based mainly on amino acid sequence identity. Genes are assigned to families when they share greater than 40% amino acid identity with reference sequences and are assigned to subfamilies when there are more than 55% identical [7]. Families are designated a CYP number based on blocks of numbers reserved for different taxonomic groups. Thus CYP51 to CYP69 and CYP501 to CYP699 are fungal families. A higher order for grouping P450 genes, called the clan has been proposed and applied to studies of P450s from different kingdoms. The introduction of clan attempts to group genes based on robust phylogenetic relationships. Genes within a clan likely diverged from a common ancestor gene [8] and may share common functions [9]. However, clan membership parameters have not been clearly defined [10]. In fungi, few phylogenetic studies using P450s have been reported. In a recent report of P450ome for *P. chrysosporium*, 12 CYP families were classified into 11 clans based on a phylogeny inferred by UPGMA [11].

In this study, we chose to characterize the P450s from four filamentous fungi for which draft sequence was recently released. We compared 2 plant pathogens, *M. grisea *(MG) and *Fusarium graminearum *(FG), both of which are classified taxonomically as Pyrenomycetes with 2 saprotrophs, *Aspergillus nidulans *(AN) and *Neurospora crassa *(NC). NC is also a Pyrenomycete whereas AN is classified as a related Plectomycete. All four fungi are Ascomycetes and share some common ecological features. All are able to grow on non-living organic matter, i.e. have a saprotrophic component in their lifecycle. However, MG and FG are distinct from AN and NC because they can also derive nutrients from living plants, behaving as parasites. In nature, MG typically infects above ground tissue, causing foliar blast disease of grasses and is particularly devastating on rice. The fungus can also infect root tissue under laboratory conditions. Its closest relatives in the Magnaporthaceae family are all root pathogens. FG is the most common causal agent of *Fusarium *head blight disease, causing sterile florets, withered kernels and mycotoxin contamination of small grains. Both FG and MG over-winter in plant debris or soil where they live as saprotrophs. AN and NC, on the other hand, are not pathogenic. AN is cosmopolitan and is commonly isolated from soil. NC is also widely distributed and typically is associated with decay of organic materials following fires. NC is notable for the discovery of an active defence mechanism called repeat-induced point mutation (RIP). RIP effectively mutates duplicated DNA sequence such as invading mobile elements and has been shown recently to be present in several other fungi including MG and some Aspergillus species. RIP has had a profound effect on the genome evolution of NC. Because gene duplication is one of the major mechanisms for the evolution of the P450 superfamily, we were particularly interested to investigate what effect RIP may have had on the evolution of P450s in NC and the other three fungal species.

To carry out the comparative analyses of P450s in these fungal genomes, all identified P450 genes needed to be first clustered in order to begin an assessment of their evolutionary relationships. However, clustering of these P450 genes was complex due to unprecedented sequence divergence. Conflicting membership of some genes to particular clusters were found when comparing results based on standard family classification and other clustering and phylogenetic analyses. In this report, we demonstrate that these conflicts can be resolved through the formulation of a standardized methodology to define high order clans. In addition, a novel approach is designed to reconstruct the P450 gene gain and loss history during the evolution of these four fungi by applying comparative studies using parsimony models.

## Results

### Distribution of P450 genes and designated gene families in filamentous fungi

**Figure 1:**
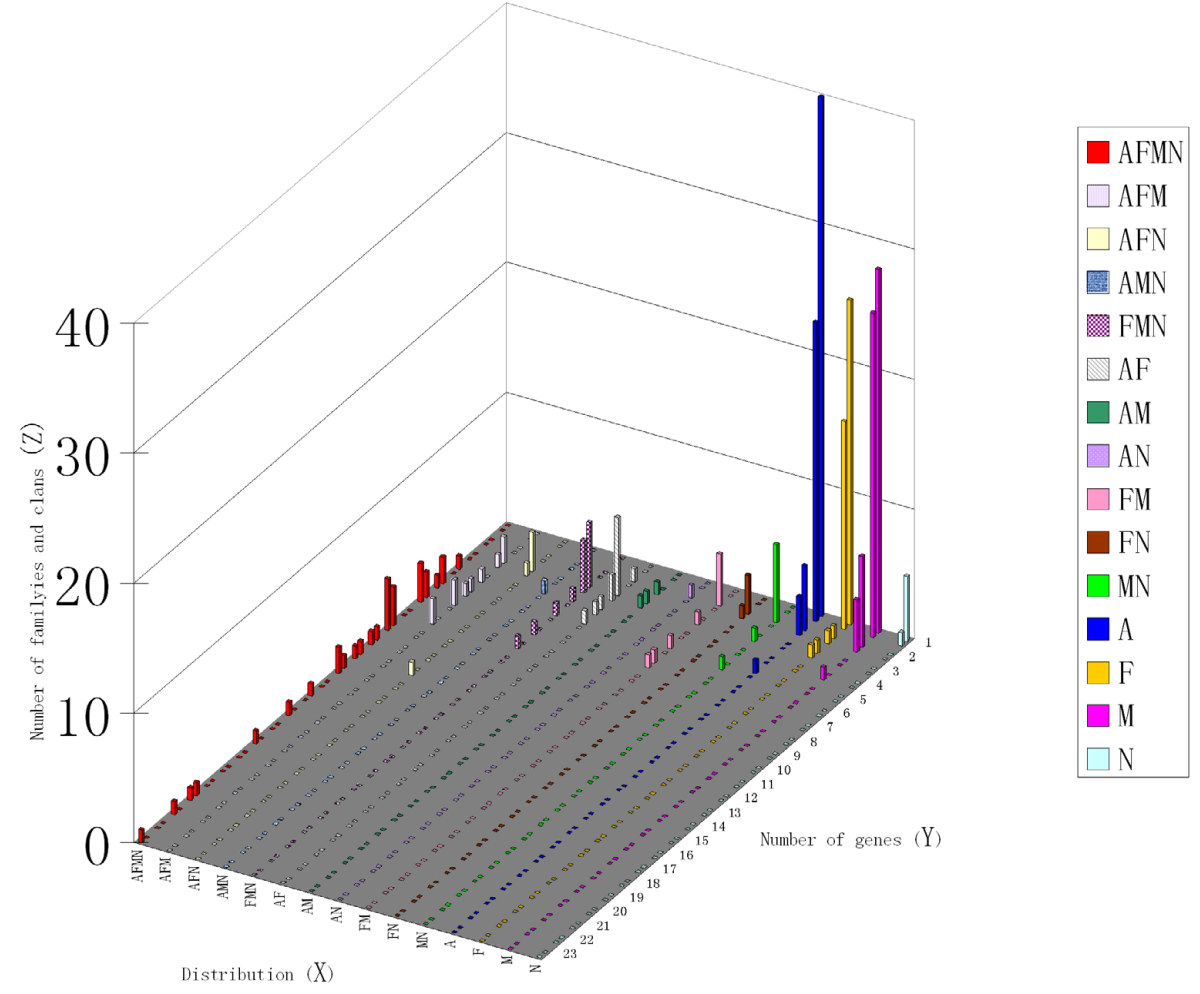
**Distribution of P450 families and clans among four filamentous Ascomycetes**. The X axis represents the combinations of the four different fungi. A refers to AN specific family or clan. F refers to FG specific family or clan. M refers to MG specific family or clan. N refers to NC specific family or clan. AFMN: family or clan whose members are present in all four fungi. AFM: families or clans whose members are present in AN, FG and MG. etc. The Y axis represents the number of genes in a particular family or clan. The Z axis refers to the number of families or clans that corresponds to each X, Y coordinate. Each X,Y coordinate contains two Z values; the distal one represents the number of families and the proximal one represents the number of clans.

**Figure 2:**
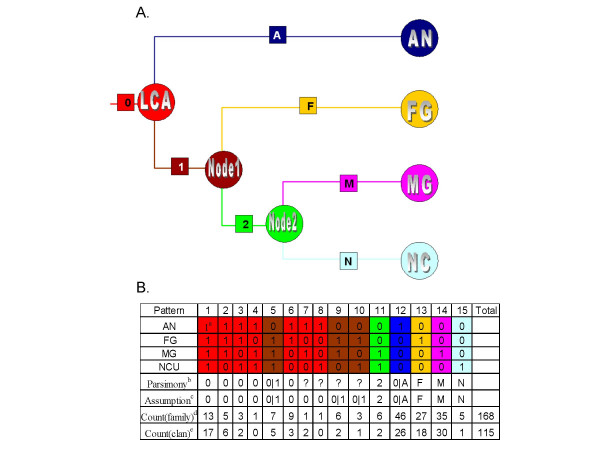
**Inference the origin of each clan or family on the branches of a species cladogram**. A. A species cladogram of the four fungi. The three internal nodes of the cladogram are named as LCA, Node 1 and Node 2 and leaf nodes are named corresponding to the four fungi. Branches connecting these nodes are named as '0,'1','2','A','F','M','N' as shown in the square embedded in each branch. Colour codes are explained below. B. Inference of the origin of standard families or clans corresponding to each of the 15 possible character state patterns. a, Number '1' in the colour area represents the presence of a certain family or clan in a fungus while '0' refers to its absence. b. The number or letters correspond to the branches shown in panel A, indicating the branch a particular pattern associated clans originated. Question mark "?" represents the origin is ambiguous as inferred by Wagner parsimony (see text for details). c. Based on the assumption that if members of a particular family or clan were present in 2 separate lineages, they were present in the LCA of those lineages (see text), the origin ambiguity were further resolved. "|" represents 'or'. d. Numbers of P450 families with particular character state pattern. e. Number of P450 clans with particular character state pattern. Colour codes: The fifteen character state patterns were classified into seven categories based on 'Assumption' and colour coded, representing the origin of family or clans associated with a particular pattern. Red: originated on branch 0; Brown: either originated on branch 0 or branch 1; Green: originated on branch 2; Blue: originated on branch 0 or branch A; Yellow: originated on branch F; Pink: originated on branch M; Grey: originated on branch N.

Inspection of the predicted open reading frame revealed that the fungal genomes contained between 107 and 122 P450s, with exception of NC which contained 41 (Table 1). In total, 381 ORFs were identified as P450 genes from the four filamentous fungi. Three were possible pseudogenes and two were less than 200 amino acids long, which were excluded from further study. For the remaining 376 genes, 322 appeared to be correctly predicted. Others were modified by the P450 nomenclature committee for possible incorrect exon calls, gene calls or fused ORFs (see Additional file 1). Based on the standard nomenclature, P450s with greater than 40% sequence identity were classified into a family. As a result, the 376 genes were assigned to 168 families. One hundred and nine P450 genes in AN were placed in 79 families, 107 genes in FG in 73 families, 119 genes in MG into 74 families and 41 genes in NC into 39 families (Table 1). Among the 168 total families, only 70 contained 2 or more members and the other 98 families contained only a single gene, i.e. were orphan families. Forty orphan families were present in AN, 25 in FG, 28 in MG and 5 in NC, respectively (Figure 1). On the other hand, only 21 families contained more than 4 genes. The largest family in this study was CYP65 which contained 20 members. However, members of this family were quite divergent in terms of the sequence identity (see below, complete linkage analyses). Furthermore, 113 families were represented in only one fungal genome, defined as self family, in a sharp contrast to only 13 families that were present in all four fungi (Figure 2). On average, a standard family contained less than 2 genes in each fungal genome. The sequence divergence of P450s in these fungal genomes was considerably more prominent than observed in animals (3~6 genes per family per genome) and plants (5~8 genes per family per genome). In summary, our results revealed that fungal genomes contain large number of P450 genes that have undergone extensive sequence diversification. Relatively few homologous gene sets are shared among the four fungi while large numbers of gene families are unique to a particular fungal genome.

**Table 1:** Comparison of genome statistics and numbers of named hypothetical Cytochrome P450 genes among four filamentous Ascomycetes, animals and plants.

	Genome size(~Mb)	ORF^a^	CYP genes^b^	CYP families	Genes/Family
*M. grisea ( *MG*)*	40	11,109	122	75	1.63
*N. crassa ( *NC*)*	40	10,082	41	39	1.05
*F. graminearum ( *FG*)*	36	11,640	107	73	1.47
*A. nidulans ( *AN*)*	31	9,541	111	79	1.41
Unique total			381	168	
*H. sapiens*	2,900	30,000	57	18	3.17
*M. musculus*	2,500	30,000	102	18	5.67
*D. melanogaster*	122	13,500	84	24	3.5
*C.elegans*	100	19,000	80	17	4.71
*A. thaliana*	129	26,000	246	47	5.23
*O. sativa*	430	60,000	345	48	7.19

a. Number of ORFs for model animals and plants are approximate.

b. Three genes from MG and two genes from AN were subsequently excluded from this study (see text for details).

### Optimization of P450 sequence alignment

Of the total 376 genes in this study, our motif finding procedure identified the heme binding signature motif in 362 genes; oxygen binding motif in 189 genes and ERR triad motif in 212 genes. Without any further adjustment in repositioning these motif sequences, we found the percentage of genes with each motif aligned correctly were 93.1%, 87.3% and 96.7% respectively. To improve alignments, the 376 sequences were split into three groups based on their signature motif alignment pattern. Group I contained 339 genes (90.2% of 376) with their signature motifs aligned well. Group II contained 15 (4%) genes whose signature motifs aligned together but were 11 amino acids downstream of the aligned position of group I. The remaining (22 genes) including the 14 P450s, which lacked a clear signature for the heme binding motif, were singleton sequences. Following building the profile alignment for group I and group II separately, the two aligned profiles were merged using profile alignment option from ClustalX. Finally, we aligned the merged alignment with the unaligned 24 sequences. As a result, we obtained an improved alignment for all 376 genes; the percentage of genes with aligned signature motif rose to 97.5% while the percentage of genes with aligned oxygen binding motif and ERR triad motif increased to 95.8% and remained at 96.7%, respectively.

### Support for standard family designation by phylogenetic and other clustering methods

NJ and MP trees were constructed using all 376 genes (see Additional file 2 and 3) and clades were collapsed into a single branch when all Operational Taxonomic Units (OTUs) in a particular clade were from one family (see Additional file 4 and 5). Analyses of these two collapsed trees revealed that out of a total of 70 families containing 2 or more genes, only 7 families did not form monophyletic groups on either tree (Table 2). It should be noted that the three families containing the largest number of genes in the four fungi, CYP65, CYP68 and CYP532, were within the list of 7. In addition, two families were monophyletic on the MP consensus tree but not on NJ tree and three families were monophyletic on the NJ tree but not on the MP consensus tree. Overall, although the grouping of most multi-member families was supported by the phylogenetic analyses, there were 12 exceptions (Table 2). This suggests possible inaccuracy in tree construction or more likely inappropriate assignment of P450s to particular families. To investigate this further, additional analyses were conducted.

**Table 2:** Assignment of P450 genes and families into clans.

clan^a^	families^b^	T^c^	B.V^d^	I^e^	UI^f^	UIP^g^
51	51(8)	8	100	12	0	0
52	52(2) 538(2)** 539(8) 584(6) 585(2) 655(1)	21	89	50	30	0.6
53	53(6)	6	100	18	14	0.78
54	54(3) 503(1) 560(1) 599(2) 602(2) 604(1) 649(1)	11	97	41	9	0.22
55	55(2)	2	100	11	3	0.27
56	56(1) 661(1)	2	87	6	2	0.33
58	58(3) 542(2) 551(2) 552(10) 681(1) 682(5)**	23	76	78	23	0.29
59	59(3)** 586(1) 587(2)~ 662(1)	7	98	21	10	0.48
60	60(2)	2	92	6	4	0.67
61	61(5)	5	100	16	1	0.06
62	62(3)	3	100	12	8	0.67
68	68(13)** 595(1) 596(1) 622(1) 650(2) 651(1) 652(1)	20	99	74	16	0.22
504	504(6)	6	100	18	8	0.44
505	505(8)^ 541(2)	10	100	27	5	0.19
507	507(1) 527(4) 535(4) 570(5)~	14	100	44	6	0.14
526	526(5) 591(1) 644(1)	7	97	17	10	0.59
528	528(3)	3	100	15	7	0.47
529	529(2) 543(2) 545(1)	5	100	13	0	0
530	530(3)	3	44	6	3	0.5
531	531(5) anCYP532E1** 631(2)	8	100	38	12	0.32
532	532(10/11)** 536(2) 629(1) 674(1) 675(1) 676(1)	16	95	60	31	0.52
533	533(2) 620(5) 621(1)	8	100	28	10	0.36
534	534(3)	3	100	13	6	0.46
537	537(4) 577(2)	6	100	28	14	0.5
540	540(9)	9	100	15	5	0.33
544	544(3)	3	100	5	3	0.6
546	546(3)	3	100	7	7	1
547	547(3)^ 581(1) 582(1) 616(1) 617(4) 618(1)	11	74	31	18	0.58
548	548(6)	6	100	18	4	0.22
550	550(3) 553(1) 633(2) 634(1) 635(1) 636(1) 660(1)	10	96	17	6	0.35
559	559(2) 606(2) 623(3) 647(1)	8	100	12	2	0.17
561	561(6)	6	100	11	0	0
563	563(2) 565(2)	4	78	15	10	0.67
566	566(3)	3	100	13	3	0.23
567	567(5)	5	93	26	14	0.54
572	572(1) 573(3)	4	100	12	6	0.5
574	574(1) 628(1) 669(1) 670(2) 671(2)**	7	70	32	6	0.19
578	578(3)~	3	85	6	6	1
593	593(2)	2	100	11	9	0.82
603	603(2)	2	100	8	4	0.5
605	605(2)	2	100	5	1	0.2
613	613(1) 686(1)	2	86	7	3	0.43
619	619(3) 665(1)	4	75	22	5	0.23
630	630(3)	3	100	5	5	1
643	643(3)	3	100	9	7	0.78
653	653(1) 654(1)	2	91	10	6	0.6
65-1	ncuCYP65B1 mgCYP65B2 ncuCYP65C1 **	3	96	16	10	0.63
65-2	fgCYP65A2 fgCYP65Q1 **	2	85	8	0	0
65-3	fgCYP65R1 mgCYP65K1 mgCYP65L1 **	3	78	8	6	0.75
65-4	mgCYP65J1 mgCYP65M1 **	2	77	5	5	1
65-5	mgCYP65F1 **	1				
65-6	mgCYP65G1 **	1				
65-7	fgCYP65S1 **	1				
65-8	anCYP65T1 **	1				
65-9	mgCYP65D1 **	1				
65-10	mgCYP65H1 **	1				
65-11	mgCYP65E1 **	1				
65-12	mgCYP65P1 **	1				
65-13	anCYP65U1 **	1				
65-14	mgCYP65un1 **	1				

a. Orphan clans (clans containing a single gene) are not listed. There are 21 orphan clans associated with character state pattern 12 (Figure 2), clan 646, 648, 656–659, 663, 664, 666–668, 672,673, 677–680, 683–685, 687; 15 orphan clans associated with pattern 13 (Figure 2), clan 506, 614, 615, 624–627, 632, 637–642, 645; 18 orphan clans associated with pattern 14 (Figure 2), clan 562, 564, 568, 575, 576, 583, 589,590,592, 597, 598, 601, 607, 608–612 and 1 clan associated with pattern 15, clan 549.

b. Parentheses indicate the number of genes within each family.

c. "T" indicates total number of genes in a clan.

d. "B.V" indicates bootstrap support for a clan.

e. "I" indicates total number of introns in a clan.

f. "UI" indicates the number of unique introns in a clan.

g. "UIP" indicates unique intron percentage. UIP = UI/I.

** Families and genes that were not monophyletic on NJ tree nor MP tree.

^ Families only monophyletic on MP tree.

~ Families only monophyletic on NJ tree.

COGs are commonly used to identify sets of orthologous genes. Our COGs analyses resulted in 80 orthologous groups (see Additional file 6). Ten groups contained members from all four fungi while 41 groups contained orthologous pairs from just two genomes. Comparing the COGs groups with the standard family designation revealed 17 groups contained members from more than one standard family. These results further support the need for efforts to classify P450 genes beyond standard family designation.

Clustering all our P450s genes was also performed based on pairwise sequence percentage identity using the complete linkage algorithm [12]. A cutoff value of 39% was found to yield the greatest degree of concordance with the standard family designation. At this cutoff, we obtained 208 complete linkage families of which 137 were identical to the standard families (see Additional file 7). Therefore, out of 168 standard families, only 31 did not cluster as one complete linkage group or contained member(s) from another family. The 31 families included 29 multi-member families and 2 orphan families. Inspection of all members of a particular discordant family revealed that the majority of multi-member families were split into several groups. Occasionally, these groups harboured members from one or more families. There were examples where a higher degree of sequence identity was observed to genes from other family(ies) than to members of its own family members, even though it formed a monophyletic group with its family members.

Overall, our results from the COGs clustering and complete linkage clustering demonstrate extensive overlap with the grouping designated by standard families. However, as noted above, members of some standard families fell into other groupings. To investigate whether these conflicts could be resolved, we decided to evaluate higher order relationships.

### Higher order grouping resolves the conflicting results from different clustering methods

To establish higher order relationships, we combined all genes belonging to one clade derived from NJ into a clan when the connecting branch was supported by bootstrap values >70%. According to Hillis and Bull [13], "70%" bootstrap value corresponds to a probability >= 95% that the clade is real. However, we recognize that equating bootstrap value with statistical significance is not universally accepted. Only two exceptions were made. The clade containing all three CYP530 genes was only supported with a bootstrap value of 44% (Table 2). However, we included them as a clan because the three genes formed a COG and they shared as high as 59% percent sequence identity (see Additional file 7). In addition, we forced the orphan clan anCYP671A1 into clan 574 that contained anCYP671B1, the only other CYP671 family member in our study. These two genes shared more than 43% sequence identity, thus forcing them in same clan conserved the original nomenclature. Overall, this resulted in 115 clans (Table 2, Figure 2); 40 were shared clans (contained members from 2 to 4 fungal genomes) and 75 were self clans (members from single fungal genomes). This revised classification reduced the self units by 38, from 113 families to 75 clans, and cut orphans (only harbouring a single gene) by 32, from 98 families to 66 clans, indicating that we were able to identify additional orthologous/paralogous relationships. Similarly, the shared standard families were reduced from 55 to 40 shared clans while 70 multi-member families were reduced to 50, reflecting a trend to cluster different families into a clan.

However, there were two exceptions to this trend. The CYP65 family was split into 14 clans and the CYP532 family was assigned into two clans (Table 2). Our new clustering schema nevertheless resolved the vast majority of conflicting results between standard family clustering and other clustering methods as described in detail below:

1). Comparison of NJ tree and MP tree with standard family designation. As shown in Table 2, seven multi-member families did not form monophyletic groups on either the NJ tree or the MP tree. As a result of formulating clans, 5 were resolved into five multi-family clans. The remaining 2 families, CYP65 and CYP532 were split. Splitting the CYP65 family was supported by both phylogenetic analyses and complete linkage clustering analysis. CYP65 members were dispersed on 7 and 10 different branches and interlaced with other family members in the NJ tree and the MP tree, respectively. Furthermore, CYP65 members were dispersed over 10 different complete linkage groups at cutoff value of 39%. In fact, the percent identity among some pairs of CYP65 members was as low as 22.5%, lower than 99.5% of all gene pairs used in this study. Therefore, it was not surprising to find that the 20 CYP65 family members were divided into 14 clans as a result of our re-classification process. As for CYP532, results of every analysis including the NJ tree (used to define clans), the MP tree and complete linkage analysis supported CYP532E1 being split from the family and merged with CYP531 and CYP631 families. Combining them formed a new clan, clan 531.

2). Comparison of COGs clustering with standard family designation. Out of the 17 orthologous groups which contained members from multiple families, 13 groups were resolved into clans after our re-classification efforts (see Additional file 6). The COG consisting of CYP614A1-CYP590A1 pair and another COG consisting of CYP665A1-CYP592A1 pair would each be clustered into a particular clan respectively if the bootstrap cutoff was relaxed slightly; 66% and 59% respectively. Thus, only 2 COGs, CYP60B1-CYP65A2 pair and the CYP611A1-CYP636A1-CYP660A1 triplet had poor bootstrap support.

3). Comparison of complete linkage clustering with standard family designation. There were 29 multi-member families and 2 orphan families which were not identical to complete linkage families formed at a cutoff of a 39% sequence percentage identity (see Additional file 7). Reducing the cutoff brought together the respective members of 15 families. All members of the other 12 multi-member families (not counting the two split families, CYP65 and CYP532) could be rejoined with their respective family members by reducing the cut-off. However, this resulted in the inclusion of members from other families. Nevertheless, each of these composite families fell into one particular clan (see Additional file 8). Finally, one orphan family, CYP545 was combined with CYP529 family members. This was also supported by our clan definition.

### Species topology of four filamentous fungi

**Figure 3:**
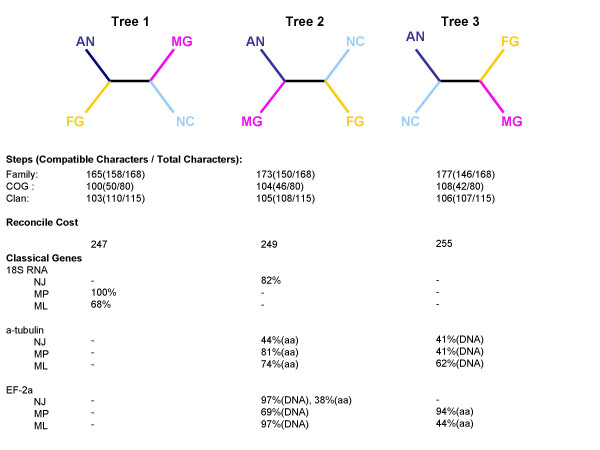
**Three possible unrooted species tree of the four filamentous Ascomycetes and their support by different analyses**. Steps refer to the fewest total number of state changes of all characters required for a particular tree topology. Compatible characters refer to the number of characters compatible with a particular tree topology (the number of steps for a compatible character equals to the minimum value for a particular tree topology compared to other topologies). Reconcile cost refers to the sum of reconcile cost for a particular tree topology as described in the text. The percentage under the classical genes refers to the bootstrap support.

For four taxa there are three possible unrooted trees (Figure 3). The species tree topology constructed by the MP algorithm based on character data matrix derived from standard family clustering, clan clustering and COGs clustering unanimously support tree 1 (Figure 3). In a recent comprehensive analysis, this tree is supported by the evaluation of 25 genes in 33 fungal species [14]. The monophyletic relationship of the Pyrenomycetes was supported by phylogenies constructed using 75 randomly selected sets of 20 concatenated genes[15]. Based on this topology, AN, a Plectomycetes, is first split from the other Pyrenomycetes. Within the three Pyrenomycetes, FG split from the common ancestor of MG and NC. This topology was also supported by results from our reconcile analyses (Figure 3). In total, 17 clans contained members from all four species. Reconciling each of the 17 genes trees to each of the three species tree topology resulted in three cost values for each clan corresponding to each species tree topology. Summing the cost value for each species tree topology across all 17 clans provided the total cost for each species tree. Tree 1 resulted in the least cost among the three topologies (Figure 3). However, species trees constructed using sequences of three classical genes, 18S rRNA, α tubulin and elongation factor 2 did not uniformly support a particular topology (Figure 3). Among the trees constructed from DNA or protein sequences of each of the 3 genes and 3 different tree construction methods, all three possible unrooted trees obtained support from at least one gene combined with one method. However, in many cases the bootstrap support was very weak. In the only three cases were bootstrap values more than 95%, two supported tree 2 and 1 supported tree 1, our preferred topology. Interestingly, in many cases using DNA sequence and protein sequence for the same gene resulted in different supported trees. Regardless of the inconsistent results using individual genes, additional analyses were conducted using tree 1, the phylogeny supported by all parsimonious analyses results using P450s.

### Reconstruction of the phylogenetic origin of clans

Among the 15 possible character state combinations representing all possible combinations of presence or absence of clans distributed within the four fungi (Figure 2), nine were unambiguous. Thus, the phylogenetic origin of 79 of 115 clans could be assigned definitely on the species tree. Clans associated with the remaining patterns had two equally parsimonious paths. Two of these, pattern 7 and pattern 8, could be resolved based on the reasonable expectation that if a clan was present in 2 separate fungal lineages, the clan was present in the LCA of the two lineages. To account for the remaining four patterns, we developed 2 parallel models; a gain and a loss model (see Methods). In the gain model, ambiguous clans were considered to be absent in LCA of the four fungi followed by a gain (0->1) event on one branch (either branch 1 or branch A of Figure 2A) and no change (0->0) on the other branch. For the loss model, the LCA were assumed to possess the clan, i.e. state 1, followed by a loss (1->0) event on one branch and no change (1->1) on the other branch.

Regardless of whether considering the gain or loss model, the number of clans gained and lost on each branch descended from Node 1, the ancestor of the Pyrenomycetes, were the same. However, the origin of some of these clans was dependent on these two models. In the gain model, some clans lost in MG or NC lineages first appeared in branch 1 (Figure 2A). In contrast, in the loss model, all clans that were lost in MG or NC were already present in LCA. A common finding between both models was that only one clan was gained in the NC lineage while 30 clans were gained in MG and 18 clans were gained in the FG lineage. On the other hand, the number of clans gained by the AN lineage was model dependent and ranged between 0 and 26. A further difference between the two models was the number of clans present in the LCA; 30 based on the gain model and an additional 34 for a total of 64 based on the loss model.

### Gene duplications and losses within each clan

**Figure 4:**
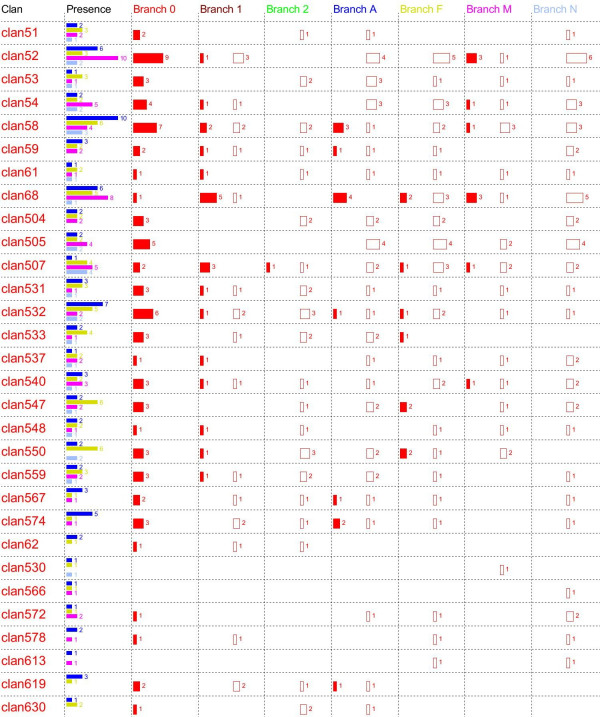
**Gene gains and losses on each branch of the species cladogram within 30 clans that were present in LCA**. The column marked "Presence" shows the number of genes of each clan present in each fungus. For the remaining columns, filled rectangles represent gene gains and unfilled rectangles represent gene losses. The number following each rectangle represents the number of gains or losses. For colour codes, see Figure 2.

The number of duplication and loss events within individual clans that occurred on all branches of the species cladogram was inferred as described in Methods. Twenty-seven clans appeared to have had at least one duplication event before AN split from the LCA (Figure 4), independent of gain or loss model, suggesting that the LCA genome contained more than one member of these clans. In total, genes from these 27 clans accounted for 103 P450 genes in the LCA genome. In particular, clans containing larger numbers of genes were usually present in the LCA. For 28 clans which contained 4 genes or more, 24 of them were unambiguously inferred to be present in the LCA. Furthermore, we found a positive relationship (correlation coefficient = 0.7) between the number of genes within a current clan and the inferred number of ancestral genes of the clan present in the LCA genome. However, one obvious exception was clan CYP68, the third largest clan. It contained 20 P450 genes from the four fungi. However, only 2 CYP68 ancestral genes were inferred to be present in the LCA genome. Thus, it appeared that clan 68 must have experienced considerable expansion after the AN split. Our data suggest that there were 5 duplication events on branch 1, 4 in branch A, 2 in branch F and 3 in branch M (Figure 4). Therefore, clan 68 is a clear example of an orthologous group of genes that resulted from filamentous fungal lineage specific expansion.

The remaining 88 clans from the total of 115 clans were predominantly small clans; only three clans contained more than 4 genes. Among these 88 clans, the number and branch location where duplication and loss events occurred could be unambiguously inferred for 54 clans. For the residual 34 clans, the number of duplication or loss events only differed between the gain and loss model at two branches; branch A and branch 1. However, the difference between the two models was exactly "1" representing either a gain of new clan or a loss of ancestral clan in either of the two branches.

### Final resolution of gains and losses

**Figure 5:**
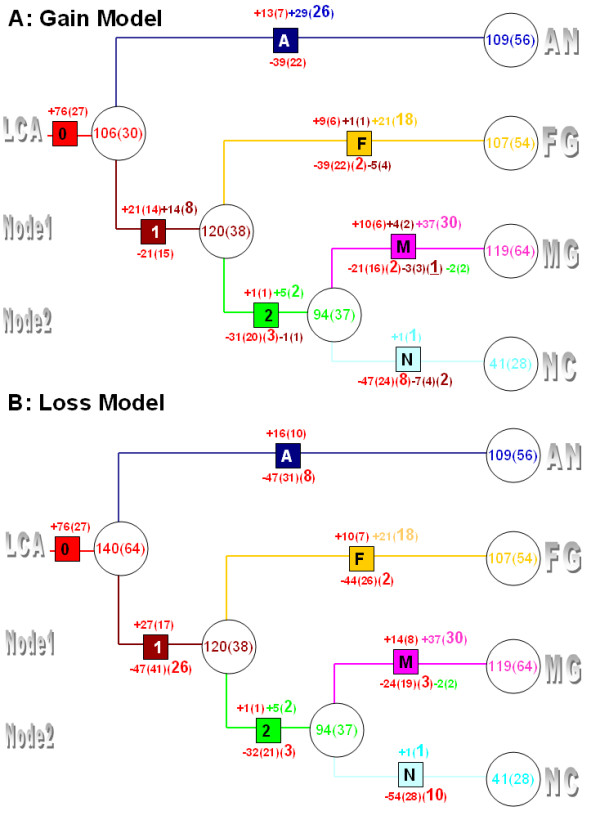
**Reconstruction P450 gene and clan gains and losses of four filamentous Ascomycetes**. Values represent number of genes gained (+) or lost (-), values in following parentheses represent corresponding number of clans. The number of new clans gained or clans lost are shown in parentheses in larger font. For colour codes see Figure 2.

Based on the inferred origin of each clan and the inferred gain or loss of genes within each clan, we deduced a complete picture of the evolutionary history of P450 genes in these four fungi (Figure 5). From the gain model, which we favour over the loss model (see discussion), the LCA genome had 106 P450 genes from 30 clans compared to 140 genes from 64 clans in the loss model. Since the gain and loss models are based on two alternative extremes, the actual number of P450 genes and clans in the LCA may lay within these boundaries. For genes belonging to the 30 clans present in the LCA based on the gain model (Figure 5A), our analyses revealed that overall loss of ancestral genes was much more prevalent than expansion. This trend was prominent on almost every branch with the exception of branch 1 where gene losses equalled gains. For example, of the 106 LCA genes belonging to 30 clans, 39 in 22 clans (1.8/clan) were lost but only 13 in 7 clans (1.9/clan) were gained in the branch A descended from LCA. Similarly, in the branch F, 39 ancestral genes derived from LCA clans were lost in 22 clans (1.8/clan) and only 9 in 6 clans (1.5/clan) gained. Moreover, branch F completely lost 2 LCA clans. NC was the most extreme example. On branch N, 47 genes derived from 24 LCA clans (2/clan) were lost and there was no evidence of gene gains. Furthermore, 8 LCA clans were completely lost in this lineage. However, this trend was more modest in branch M. Only 21 genes in 16 clans (1.3/clan) were lost, 10 genes in 6 clans (1.7/clan) were gained and 2 LCA clans were completely lost. In sum, gene losses from the LCA derived clans appear to have occurred much more frequently than gene gains on all branches descended from Node1 as well as on the branch A during evolutionary history, regardless of model. The rate of gene duplication and loss within clans was similar for three of the fungal lineages, AN, FG and MG, ranging between 1.3 and 2 events per clan in the gain model. In the NC lineage, on the other hand, the LCA derived genes were exclusively lost.

In addition to gene duplication and loss events from within LCA derived clans, a large number of new clans were created in each fungal lineage with the exception of NC. In the NC lineage branch (branch N), there was only evidence for the gain of a single new clan (one gene), whereas AN, FG and MG gained 26 (29 genes), 18 (21 genes) and 30 (37 genes) new clans, respectively (Figure 5A). These lineage specific gains occupy a significant part of current P450omes in AN, FG and MG. They also appear to compensate for gene losses that occurred from within the LCA clans so that the current P450omes of the three fungi are approximately similar in size to the predicted number present in the LCA. NC is an obvious exception and has much smaller P450ome compared to the LCA and the other three fungi. This appeared to be the result of losses of ancestral P450 genes coupled with only a single gene gain.

Our observations above are in general consistent when the loss model is applied (Figure 5B), the only major exception being that the LCA genome would possess a much larger P450ome (140 genes in 64 clans). Another consequence of applying the loss model is that there would be no lineage specific gains and more contraction of the LCA clans on branch A and in the branch 1. Therefore, invoking the loss model as the other extreme does not compromise but actually strengthens our conclusion that the LCA derived clans experienced significant loss events and few gains. Furthermore, the total number of gains and losses were the same regardless of whether applying either model on all branches descended from Node 1, the ancestor of the Pyrenomycetes.

Evaluating the overall gene gains and losses on each of the four fungal lineage specific branches, regardless of origin, revealed that MG lineage gained nearly twice as many genes compared to losses. This is in complete contrast to FG, AN and NC. The MG lineage branch gained 51 genes and lost 26, whereas the FG lineage branch gained 31 and lost 44 and AN gained 42 and lost 39 in the gain model and gained 16 and lost 47 in the loss model. The NC lineage only gained 1 and lost 54. The large number of gene gains in the MG lineage is intriguing because this lineage presumably evolved more recently, particularly compared to the AN lineage.

### P450 genes do not appear to be highly clustered in filamentous Ascomycete fungal genomes

**Figure 6:**
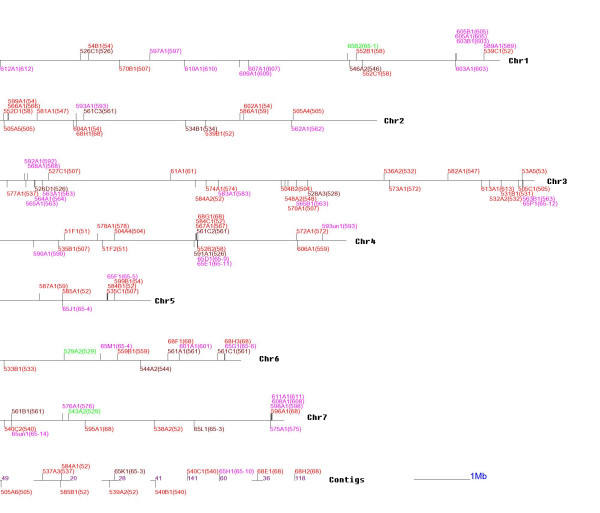
**The physical location of P450 genes in the *Magnaporthe grisea *genome**. Each horizontal line represents a chromosome. Those at the bottom followed by a number in purple colour represent unanchored contigs. A vertical bar above (on 5-3' strand) or below (3'–5' strand) horizontal line marks the position and orientation of each CYP gene, the name of which is marked by omitting the leading "CYP". The number after each gene in parenthesis represents the clan to which this gene was assigned. For colour codes, see Figure 2.

We defined a gene cluster as 4 or more P450s present within a 100 kb sliding window of genome sequence. This revealed the presence of 3, 3, 3 and 0 P450 gene clusters in the genomes of AN, FG, MG and NC, respectively (Figure 6 and Additional file 9). However, none of the clusters contained all P450 genes from the same family or clan. At most, two genes were from the same clan or family in any gene cluster. Furthermore, the gene pairs which resulted from lineage specific duplication events, as inferred by our gain model, were not located within the same clusters.

### Intron-exon structure is marginally conserved

Among the 376 P450 genes, 15 contained no introns. In contrast, CYP659A1 from AN had 24 introns, while the next highest number of introns in a P450 gene was 12. The remaining P450s harboured between 1 and 10 introns (see Additional file 10). The average number of introns present in each gene was 3.2 and totalled 1207. As described above, P450 genes were associated with 50 multi-member clans and 65 orphan clans. Nine hundred and eighty-six (986) introns were present in the 311 genes belonging to multi-member clans. A string of numbers representing the intron-exon structure and Unique Intron Position (UIP) value for each clan are shown in Table 2 and Additional file 11. The results demonstrate that the intron-exon structure is not well conserved for most clans. First of all, only 16 multi-member clans (only 4 of them contained more than 3 genes or more) had at least one consensus intron (an intron shared by all members of a clan at the same aligned position). There were a total of 28 clans with more than 3 members, only 4 had at least one consensus intron. Secondly, 39% (383) of the total 986 introns were unique (intron occupying a unique position in the alignment). The remaining 606 introns were present in 179 positions among the 50 clans. Therefore, it was not surprising to observe a UIP value as high as 0.45 on average, across all clans. Thus approximately half of the introns within each clan were unique. However, the UIP value varied greatly among clans. There were 4 clans with a UIP value 0 (no intron occupied a unique position) as well as 4 clans with a value 1 (all introns occupied a unique position) (Table 2). Values from clans other than these eight exhibited approximately a normal distribution with a mode value around 0.5 (see Additional file 12). Therefore, intron-exon structures were not well conserved for most P450 clans with a small number of apparent exceptions. When considering individual genomes of the four filamentous fungi, the UIP value for the majority of clans were also high with consensus introns being rare. For example using the largest clan, clan CYP58, consisting of 23 genes (see Additional file 13), the UIP was 0.43 for all 32 introns in 10 AN P450 genes in this clan, 0.22 for 22 introns in 6 FG P450 genes, 0.20 for 11 introns in 5 MG P450 genes and 0.60 for 13 introns in 3 NC P450 genes, as compared to an overall UIP value for this clan of 0.3.

## Discussion

### P450ome of filamentous ascomycetous fungi exhibit considerable divergence in terms of sequence, intron-exon organization and genome location

In this work, we discovered that filamentous ascomycetous fungi, with the noticeable exception of NC, contain in excess of 100 cytochrome P450 genes. This is close to the number found in the basidiomycetous fungus (*P. chrysosporium*), animals (human, mouse, *D. melanoganster *and *C. elegans*), but considerably fewer than found in plants (*A. thaliana *and *O. sativa*). Overall, between 0.2–1.1% of the entire predicted open reading frames encode P450s. A notable difference between the P450s in the filamentous Ascomycetes and other organisms is the level of sequence divergence. P450s in the fungi studied here are considerably more divergent. With the exception of NC, P450 genes within filamentous Ascomycetes fall into more than 70 distinct families, each possessing ~1.5 genes on average, whereas for plants and animals, the number of distinct families ranges between 17 and 48, with each family containing between 3 to 7 members on average. In NC, each family contains a single member with only 2 exceptions (CYP527 and CYP68 families). NC possess a genome defence mechanism, known as RIP which serves to destroy repeat DNA sequence such as invading viruses [16]. This mechanism presumably was able to effectively suppress creation of new genes via gene duplication. In contrast, *P. chrysosporium *has been reported to contain relatively few distinct families, each contained 10 members on average [11]. In plants, animals as well as *P. chrysosporium*, intron-exon organization is well conserved [11,17,18]. For example, the A-type (plant specific) P450 clade in *Arabidopsis *which contains 72 genes and several families has very few unique introns (UIP value <0.1) and forms a single monophyletic group. In addition, there is one clearly conserved intron present at the same position of almost all of the genes (group consensus intron). This is significant because half of the genes have only one intron while the others possess at most three introns with one exception. This is in distinct contrast to our findings for filamentous Ascomycetes. The vast majority of clans containing multiple members did not contain a conserved intron, and nearly half of the introns were unique. It is noteworthy that the intron positions in *P. chrysosporium *members of two well studied families (CYP63 and CYP505) were generally well conserved and co-localized in the genome [11]. This suggests that gene duplication typically occurred locally and that these events occurred relatively recently. In contrast, there is little evidence of co-clustering of family members in the genome of filamentous Ascomycetes, suggesting they are more ancient as evidenced by sequence divergence and that they have become widely distributed about the genome predominantly through translocation events [19]. With more sequenced fungal genomes being sequenced, we are currently investigating whether it is generally true that Ascomycetes tend to have a much more diverged P450ome than basidiomycetes. If this turns out to be true, does this trend apply to other gene families?

### Need for higher order classification of fungal P450 genes

Our attempt to reconstruct the phylogeny for P450s in filamentous Ascomycetes was hampered by their extreme sequence divergence and the large number of genes involved. To initiate phylogeny reconstruction, we identified the three core sequence motifs as an aid to ensure the best possible alignment of the sequences. The motif logo was useful for the signature motif, but due to sequence divergence, our ability to define good sequence logos for the other motifs was limited. We also used introns-exon structures to check the accuracy of our final alignments. As shown in Additional file 11, intron position generally aligned well in the few cases where they were conserved. Attempts to reconstruct the overall phylogeny using the aligned sequences by distance (NJ) or parsimonious (MP) method were unsuccessful due to conflicting relationships obtained by the different methods. However, by proposing a higher order grouping, the clan, the vast majority of these conflicts evaporated. Moreover, the clan groupings were generally very well supported by both COGS and complete linkage clustering.

The concept of "clan" was proposed to accommodate the flood of new P450 families designated by the standard nomenclature committee. P450 clans were proposed to encompass genes derived from a single common ancestor and could contain one or more families [8]. However, previous efforts to define P450 clans in plants and animals are somewhat arbitrary and clan classification has not been standardized. Consequently, ten and nine clans were proposed to occur in plants and vertebrates respectively [20-22]. In order to formalize the clans for Ascomycete fungal P450s, we selected groupings that were supported by a bootstrap value of >= 70%. This value has been adopted by many systematists as an indication of support for a particular topology [23]. Using the 70% cutoff value on a phylogenetic tree, we found that the vast majority of conflicting clustering results from the different methods used in our studies were resolved. This cutoff maybe too stringent in some instances to define clans because there were several examples of where pairs of orthologous genes were clearly grouped together on the phylogenetic tree with bootstrap value of <70%. However, making these assessments requires considerable manual inspection.

### A novel strategy to trace the ancestral history of genes in a highly divergent superfamily

After assigning P450s into clans, we wished to identify the ancestral origin of each clan and to explain the current distribution of genes in the four fungal species on a clan by clan basis. An approach to begin to address these questions is to view each clan as an evolving character in the hierarchical context of the species tree [24]. As described in results, there is not universal support for one particular species topology for the 4 fungal species used in our study, however, most evidence points to the tree we settled on for our evolutionary reconstruction studies (Tree 1, Figure 3). This topology is supported by our P450 data as well as a recent report from Galagan et al [14], which used 33 fungi, including the 4 used in our studies, and protein sequences from 25 genes. To reconstruct the phylogenetic origin of P450 clans, we applied Wagner parsimony to infer the ancestral state, which assumes gain and loss are equally parsimonious. A common assumption was made that clans present in two descendents were inherited from their last common ancestor in order to resolve some paths that were equally parsimonious. In other cases where ambiguity could not be resolved, two models were proposed, a gain model and a loss model. The number of character state changes resulting from application of these two models likely represents the two extremes.

Parsimony analysis using the reconcile process was used to explain the current distribution of P450 genes within clans for the 4 fungal species superimposed upon the species tree. Identification of the duplication and loss events is straightforward in the clans that exhibited a single most parsimonious path when inferring their origin. However, for clans that originated in branches descending from the LCA node, it was necessary to impose a gain event at the branch where the clan originated. On the other hand, for clans whose origination was ambiguous, the gain or loss model needed to be inferred prior to applying the reconcile process.

Gain and loss models were introduced to account for the parsimonious ambiguity for inferring the origin of certain clans. Comparing the virtues of the gain and loss models, we believe the gain model more closely represents the real world situation for the following reasons. First, the overall branch length in the gain model appears to be more compatible with species evolution than the loss model, assuming the branch length is proportional to the sum of clans (or genes) gained and lost by each lineage. For example, in the gain model, 26 new clans appear in the AN lineage and no loss events from the LCA. In the FG lineage 26 clans were gained and 2 were lost for a total of 28 from the LCA (see Figure 5). Similar values would be expected because the same period of time has elapsed from when the Pyrenomycetes split from the Plectomycetes. In contrast, in the loss model, the AN lineage gained no new clans and lost 8, whereas the FG lineage gained 18 and lost 28 for a total of 46. Furthermore, under the loss model, it would not seem reasonable that while FG and MG lineages gained new clans none appear to be gained in the AN lineage.

It is worth noting that the number of clans and genes present in the ancestor of the Pyrenomycetes (Node 1) and the total number gained and lost on the descendent branches are not model dependent, suggesting that the evolutionary history of these P450s are robustly predicted.

### The evolution of P450s in filamentous Ascomycetes

By comparing the number of genes under both models, the LCA genome may have possessed between 106 to 140 P450 genes. The LCA thus had a similar size of P450ome compared to the current filamentous Ascomycetes except for NC. Thus, the P450ome size does not appear to have undergone expansion for around 6 hundred to 7 hundred Million years in the Ascomycete branch within the tree of life [25]. However, based on the gain model favoured by us as discussed above, the P450 genes in the LCA were classified into 30 clans compared to the >50 present in AN, FG and MG. In particular, regardless of gain or loss model, the last common ancestor of the three Pyrenomycetes (Node 1) contained 36 clans, considerably fewer than present in FG and MG. Thus, during the evolution of these fungal lineages the P450ome has continued to diverge with the apparent loss of members from these 30 clans and the emergence of genes in new clans. It is intriguing to speculate whether there exists a certain selection pressure or stabilizing requirement which prohibited an overall gain in the size of the P450ome during the evolution of these four fungi. Also, whether there is any selection pressure to maintain a balance between these 30 conserved clans and those that newly emerged. These 30 clans may represent the minimum P450ome of filamentous Ascomycetes as most of them are maintained in the four fungi (AN retains all 30 while FG lost 2 and MG lost 2, whereas NC with a much smaller P450ome, maintained 22 of these clans). These conserved clans may represent the core functions of P450s required by filamentous Ascomycetes and most are likely present in other related filamentous Ascomycetes. Notably, eight out of the total eleven P450 clans in the basidiomycetous fungus *P. chrysosporium *[11] overlapped with these core clans. These core clans usually contained multiple members (average 3.3) in the LCA. Thus the loss of entire clan in other fungal lineages after the LCA would require multiple loss events of ancestral genes, which is unlikely and would be probably selected against. Ancestral genes in these clans were gained and lost at a very similar rate until the FG lineage emerged. During this same period, several new clans appeared. Thereafter, but before the emergence of the MG and NC lineages, loss instead of gain dominated the evolution of genes with respect to these core clans. In the NC lineage, loss of P450 genes from core clans continued. In the MG lineage, there was expansion of some of the core P450 clans accompanied by the appearance of a large number of new clans. As described in results, it is perhaps not surprising that the NC lineage was unable to evolve new clans and that genes were lost from ancestral clans due to the presence of RIP. However, it is interesting to note that regardless of invoking the gain or loss models that there were very few genes gains to new or core clans immediately prior to the MG and NC split. This suggests that the ancestor common to MG and NC may also have had a mechanism to eliminate duplicated genes. Indeed, a RIP-like mechanism has been reported in MG and several other fungi including Aspergillus species [26]. However, RIP is not active in all MG strains and appears to be much milder than observed in NC. Other factors, nevertheless, such as selective pressure, ecological niche adaptation may also have driven P450ome evolution. For example, after NC and MG lineage split, the expansion of P450ome in the MG lineage may have resulted from increased selection pressure to successfully adapt to its pathogenic life style on a number of grass host species. In contrast, it is possible that P450 gene expansion would interfere with or provide no advantage for NC to survive as a saprotroph.

For clans which we were unable to reliably assign origin to the LCA, they may have been either derived from a gene which was horizontally transferred, such as the CYP55 clan, or diverged to the extent that we can not trace their origin to a core clan. Genes in CYP55 clan, such as P450norA and P450norB from *Fusarium oxysporum*, which encode nitric-oxide reductase, have been shown, based on sequence comparison [27] and phylogenetic analyses [28], to be more closely related to bacterial genes such as streptomycetes CYP105 and CYP107 than to eukaryotic P450s. In the latter and more typical instance, members of new clans likely represent genes which experienced neofunctionalization following duplication from the ancestor P450omes. These genes probably evolved at a fairly fast rate after duplication while the selective constraint was relaxed. Thereafter, there may have been a period of positive selection, which resulted in neofunctionalization and their retention in the genome. Our parsimonious analyses revealed that 31% of the MG P450ome, 20% of FG P450ome and 2% of the NC P450ome were derived from clans created within their own lineage. For AN, 26% were derived from its own lineage based on the gain model. Therefore, the MG lineage appears to have experienced a higher degree expansion of new clans compared to the other fungi in our study. Additionally, the contraction of core P450 clans appears milder in MG compared to the other three. The biological relevance of these events is unknown; however, it may suggest that the MG has been involved in more intense warfare with its hosts than the other plant pathogen, FG, and/or MG has been more active in exploiting diverse habitats than the other three fungi. Detailed analyses including functional studies of specific clans in these four fungi that exhibit differential expansion or contraction are currently under investigation.

## Conclusion

Our understanding of the processes that fungi evolved to exploit a variety of ecological niches is limited but of fundamental biological importance. Among all gene families, the P450 superfamily likely plays an invaluable role. The four fungal genomes we studied contained a relatively large number of P450 genes. Although, it is known that this superfamily exhibits considerable divergence, the level of divergence in ascomycetous fungi appears to be unprecedented. The degree of divergence greatly hampered our ability to apply advanced or traditional phylogenetic models to build the phylogeny for these genes, and required us to develop a novel strategy. To define more robust gene sets, each possibly derived from a single ancestral gene, we compared the standard family classification with results from other clustering methods. By proposing a standardized clan with 70% bootstrap support, we successfully resolved conflicting results from a variety of analyses. Combined with applying parsimonious principles, we are able to construct a model which more clearly explains the evolutionary path of P450 genes in the four fungi used in our study. We identified 30 clans which may represent the core functions of ascomycetous fungi and found that the contraction of genes in these clans was more prominent than gene gains. To compensate in some way, a large expansion of new clans was found in three fungal genomes, the exception being NC, which was perhaps predictable based on the presence of the RIP mechanism. Intriguingly, this expansion was even more prominent in MG lineage. These results combined with our other findings such as the prominent lack of conservation of intron-exon structure for P450 genes and little clustering of homologous genes in the genome, suggest the evolutionary history of ascomycetous fungal P450ome is not simple and may include rapid sequence evolution and genome evolution. It is possible that this rapid and complicated evolutionary history may be an essential feature for fungi to have emerged as highly successful inhabitants of planet earth.

## Methods

### Identification of P450s

Release 3, Release 2, Release 4 and Release 3 of predicted open reading frames for *Aspergillus nidulans*, *Fusarium graminearum*, *Magnaporthe grisea *and *Neurospora crassa *respectively were downloaded from the Broad Institute [29]. InterProScan v3.1 package [30] was downloaded from the European Bioinformatics Institute website and run on the coding sequence dataset for each fungus. Cytochrome P450 genes were identified containing the InterPro domain IPR001128.

All predicted P450 genes were submitted to the P450 nomenclature committee [7] for confirmation, assigning a CYP name which provides a "standard" family and subfamily designation for each particular gene. Possible pseudogenes or genes with less than 200 amino acids were excluded from further analyses.

### Orthologous groups clustering and complete linkage clustering

1). Orthologous groups:

Orthologous groups were formed following the COGs (Clusters of Orthologous Groups) construction protocol [31] with minor changes. Specifically, sets of P450s from each genome were reciprocally blasted [32] in all combinations of genome pairs using BlastP program with an E value cutoff of 1E-50. Genome-specific reciprocal best hits (BeTs) between two genes from two genomes were considered a COG. Three genes from different genomes were linked together to form a COG when all pairwise comparison resulted in BeTs. For 4 genes to be considered a COG, at least 5 of the 6 pairwise comparisons had to result in BeTs.

2). Complete linkage clustering:

All pairwise combinations of P450 genes were aligned using amino acid sequences by the SSEARCH program [33] of the FASTA package (Pearson, W. R). SSEARCH was run using the BLOSUM50 matrix with gap open and extension penalties of -3 and -1, respectively. P450 genes were then clustered using the complete linkage algorithm of the OC cluster analysis program [12] based on the sequence identity of all pairwise genes reported from SSEARCH.

### Identification of P450 conserved sequence motifs

Typically, P450s contain three sequence motifs. The P450 heme binding signature motif was identified by searching for the sequence motif 'F**G***C*G'(* represents any amino acid). Where no such sequence pattern was found, we carried out a two-round search for variants. For the first round, we searched for variants including '***G***C*G' or 'F******C*G' or 'F**G***C**'. The search concluded if at least one variant was found. Otherwise, we went to a second round of searching for variants including 'F******C**' or '***G***C**' or '*******C*G'. When more than two signature motifs were found, the one which resided closest to the C terminal was selected. The P450 ERR triad motif was identified by searching for the sequence motif "*E**R*+PER*" (+ represents variable length of amino acids). If more than two ERR triad motifs were found, the one whose length was closest to 50 amino acids and at the N-terminal side of signature motif was chosen. P450 oxygen binding motif was identified by searching for the sequence motif '[A/G]G* [E/D]T [T/S]'([/]represents either or). If more than two oxygen motif motifs were found, the one closest to the N-terminal side of ERR triad motif was selected.

### Sequence alignment

All multiple sequence alignments in the study were constructed using ClustalW [34]. For multiple sequence alignment of all P450 genes, several steps were applied based on the aligned positions of the three motifs (P450 signature motif, ERR triad and oxygen binding site). The protocol involved: 1), a multiple sequence alignment of amino acid sequences for all genes using ClustalW with a gap penalty of 10, a gap extension penalty of 0.05 and Blosum as protein matrix series; 2), marking the start position of the three motif sequences on alignments; 3), where the signature motifs were not aligned, genes were divided into groups so that each group consists of genes whose signatures motifs were aligned together; 4), perform multiple sequence alignment for each group; 5), perform profile alignment combining all groups' alignment profiles and 6), removal of N and C terminal columns when less than 2 amino acids in data columns.

### Phylogenetic tree reconstruction and assignment of genes to clans

Neighbour Joining (NJ) including bootstrapped trees and Maximum Parsimony (MP) trees were generated using MEGA3 [35]. Multiple substitutions were corrected using the Poisson distance model. Alignment gaps were treated by choosing pairwise deletion option for the NJ method. Close-neighbour-interchange algorithm was used to find the MP trees. Consensus trees were generated using Consensus Program of Phylip v3.6 package [36]. Monophyletic clades in both the NJ tree and the majority consensus tree of all MP trees whose Operational Taxonomic Unit (OTU) were from same family were collapsed if they contained members from the same family. Bootstrap tests of 1000 trials were carried out for the NJ tree. Each monophyletic clade supported by a bootstrap value > 70% was designated as a new gene unit, defined here as clan.

### Inferring gene gains and losses

Gene gain and loss events in the whole evolutionary history of the four fungi were constructed in a two step procedure. 1. Infer origin of each clan. 2. Infer gene duplication and loss events within each clan.

To infer the origin of each clan in the evolutionary history of these four fungi, a two-dimensional matrix was first built using discrete characters 1 and 0, representing the presence or absence of member of a predefined clan in each fungus. Each family or clan was assigned to one of a total of 15 ((^4^_4_) + (^4^_3_) + (^4^_2_) + (^4^_1_)) possible character state combinations. The Mix program of Phylip v3.6 package was then applied to infer the most parsimonious species cladogram as well as the origin and subsequent state change of each character. The Wagner parsimony method was chosen to allow 0->1 change and 1->0 change with equal weight. "Print out steps in each character" and "Print states at all nodes of the tree" options were chosen to view the state change process for each clan. To account for the ambiguous origination of some clans, one assumption and two alternative models were proposed. The assumption was that clans defined by us represent truly orthologous or paralogous groups. Thus, for clans that contained genes from different fungal species, the common ancestor of these fungal species contained the ancestor gene of this clan. To explain clans whose origination was not resolved after applying the assumption, a gain model and a loss model were proposed simultaneously. For the gain model, gain (0->1) is more possible than loss when two equally parsimonious paths existed and verse visa for the loss model.

To infer gene duplication and loss events within each clan, reconciling gene tree of each clan to species tree was carried out using GENETREE 1.3 [37]. To obtain a gene tree for each clan, Maximum Likelihood phylogenies with bootstrap were generated using Seqboot, Proml and Consensus program of Phylip v3.6 package with the JTT probability model. The diatom CYP51C1 gene sequence was used to root all gene trees. A member of the CYP51 was selected because CYP51 formed a distinctive evolutionary cluster independent of other P450 families and all "extant" CYP genes in eukaryotes were presumably derived from this family [38-40]. The species cladogram obtained from step 1 was used as the species tree input for GENETREE. Specifically, three cases needed to be taken into consideration. First, for clans that originated before the last common ancestor (LCA) of the four fungi (see LCA in Figure 2A), gene gain and loss events were read directly from the reconciled trees. Secondly, for the shared clans (contained members from 2 to 4 fungal genomes) whose origination was after the split of AN from LCA, one additional duplication event was required to be added in the branch corresponding to the gain of the new clan. Thus, the gene trees of these clans only needed to be reconciled with the corresponding 3-taxon species tree (FG, MG, NC) for clans originated on branch 1 (see branch 1 in Figure 2A) or 2-taxon species tree (MG, NC) for clans that originated on branch 2 (see branch 2 in Figure 2A) to infer the following gain and loss events after the gain of the clan. Thirdly, for clans that originated specifically in each of the AN, FG, MG or NC lineages, the number of duplication events for this clan in the corresponding fungal lineage equals the number of genes. Final models representing the reconstructed gain and loss events laid upon the phylogeny of the four fungi were derived by combining results from step 1 and step 2 as described.

### Species phylogeny

The species cladogram of the four filamentous fungi was studied using three different approaches. First, we derived the maximum parsimonious species topology using discrete characters data consisting of 1 and 0 representing presence and absence of a certain "group" in each fungal genome. The group data was derived from 1). standard families 2). COG groups and 3). groups based on our defined clans. The Mix program of Phylip v3.6 package was implemented with Wagner parsimony. Secondly, we used data from the reconciled gene trees of several clans to create species trees. Specifically, we chose those clans whose members were present in all fungi and built the gene tree phylogeny for each of them. The cost of embedding a gene tree for a clan within a species tree (three possible topologies) was calculated by the Genetree program. We then summed the reconcile cost of all clan gene trees for a particular topology to calculate its overall cost. The tree with the lowest cost was considered to most parsimonious. Finally, we used three classical genes, 18S rRNA, elongation factor-2 and α tubulin to build the species phylogeny. Both DNA sequence and encoded protein sequence were used for elongation factor-2 and α tubulin. In addition, NJ, MP and Maximum Likelihood methods were applied using the protocol as described for constructing the phylogeny for P450s.

### Genome distribution of pattern of P450 genes

The versions of genome assembly used to map P450 genes on four fungal genomes were: Release 4 for MG; Release 2 for FG; Release 3 for An; and Release 3 for NC. The location of P450 genes were mapped to their respective contigs, supercontigs with orientation noted. The positions of P450s in each genome were drawn by a custom Perl script.

### Intron-exon structure

Intron and exon structure information for each P450 gene was downloaded from Broad Institute [29]. For some genes whose coding sequences were revised by the Standard Nomenclature Committee, the intron-exon structures were revised in accordance. Intron-exon structure divergence between two genes was quantified as the number of discordant introns (introns in one gene without counterpart intron in the same position and same phase in other genes). To detect discordant introns between two genes, sequences were aligned first using ClustalW, followed by marking the intron positions and then comparing the position and phase of all introns between genes. For a clan of P450s, the intron-exon structure pattern was presented by a number string separated by '-', which summarized how many introns were shared by how many clan members, i.e. the first number in the pattern represented the number of group consensus introns (introns present in all group members because of same aligned position). The second number following the separator '-' represented the number of introns present in all but one gene. The third number following the separator '-' represented the number of introns present in all but two genes, etc. The last number in a pattern represented the number of singleton introns in a group. In addition, we propose here Unique Intron Proportion (UIP) to account for the dissimilarity of the intron-exon structures among a group of P450 genes. UIP is the proportion of unique introns (introns present at a unique position in the alignment). Groups with higher UIP values indicate higher proportion of unique introns and thus are less conserved for their intron-exon structures.

## Authors' contributions

JD set up the database, wrote all computer codes, carried out the detailed analyses, and wrote the draft manuscript. IC was involved in curating the sequence alignment, the phylogenetic analyses, and helped with the introduction and application of different methodologies to analyze the data. RD conceived the study, was responsible for overall coordination and preparation of the manuscript. All authors helped with data interpretation and revision of the manuscript.

## Supplementary Material

Additional file 1 **All P450 genes, cds, cds length and locus name in *A. nidulans*, *F. graminearum, M. grisea *and *N. crassa *as annotated by the Broad Institute.**. a. "Same" indicates the coding sequence (cds) used in the study exactly matches the locus predicted by the Broad Institute. "Include" indicates the cds in this study corresponds to a locus predicted by the Broad Institute where one or more exons were modified. "Not includ" indicates cds excluded from this study. b. Locus as defined by the Broad Institute Click here for file

Additional file 2 NJ tree of 376 P450s from 4 filamentous Ascomycetes Click here for file

Additional file 3 MP tree of 376 P450s from 4 filamentous Ascomycetes Click here for file

Additional file 4 **Collapsed NJ tree of 376 P450s from 4 filamentous Ascomycetes**. Each collapsed branch is followed by a number representing the CYP family taxon name. The number in <> after the CYP family name represents the number of genes under this collapsed branch. Number after the ...represents the total number of branches containing other members of the same CYP family in this collapsed phylogeny. Click here for file

Additional file 5 **Collapsed MP tree of 376 P450s from 4 filamentous Ascomycetes**. Each collapsed branch is followed by a number representing the CYP family taxon name. The number in <> after the CYP family name represents the number of genes under this collapsed branch. Number after the ...represents the total number of branches containing other members of the same CYP family in this collapsed phylogeny. Click here for file

Additional file 6 **Orthologous groups identified by COGs analyses**. # indicates COGs containing genes from a different P450 family. * indicates CYP636A1 and CYP660A1 were assigned to clan550 while mgCYP611A1 was excluded. ** indicates bootstrap support <70% but >50%, see text for details. Click here for file

Additional file 7 **Complete linkage families formed at cutoff of 30% of sequence percentage identity**. * refers to complete linkage families which were identical to standard families. Click here for file

Additional file 8 **Standard families not identical to complete linkage families are resolved to clans**. # indicates standard family was split into different clans (see text). $ indicates family rejoined to a group exclusively consisting of this family members. Click here for file

Additional file 9 **The physical location of all P450 genes in AN, FG and NC genomes**. Each horizontal line represents a chromosome. Those at the bottom followed by a number represent unanchored contigs. A vertical bar above (on 5-3' strand) or below (3'-5' strand) horizontal line marks the position and orientation of each CYP gene, the name of which is marked by omitting the leading "CYP". The number after each gene in parenthesis represents the clan to which this gene was assigned. For colour codes, see Figure 2. Click here for file

Additional file 10 Distribution of the number of intron for each P450 gene among all 376 P450 genes Click here for file

Additional file 11 **Intron-exon organization pattern derived from two alignments**. Clan alignment: alignment based on genes belonging to individual clans. All alignment: alignment of all 376 genes as described in text. Click here for file

Additional file 12 **Distribution of UIP values among multi-member P450 clans**. UIP value of each clan was assigned to 10 intervals (0–0.1; 0.1–0.2; 0.2–0.3; ...0.9–1). Click here for file

Additional file 13 **Intron-exon organization of clan 58**. Horizontal lines represent P450 amino acid sequences. A vertical bar on the horizontal line represents an intron. The number under the bar represents the intron phase. Click here for file
