# Supplementary figures and images for: The evolutionary history of Cytochrome P450 genes in four filamentous Ascomycetes
Source: BMC Evol Biol. 2007 Feb 26;7:30. doi: 10.1186/1471-2148-7-30 (PMC1828051; doi:10.1186/1471-2148-7-30)

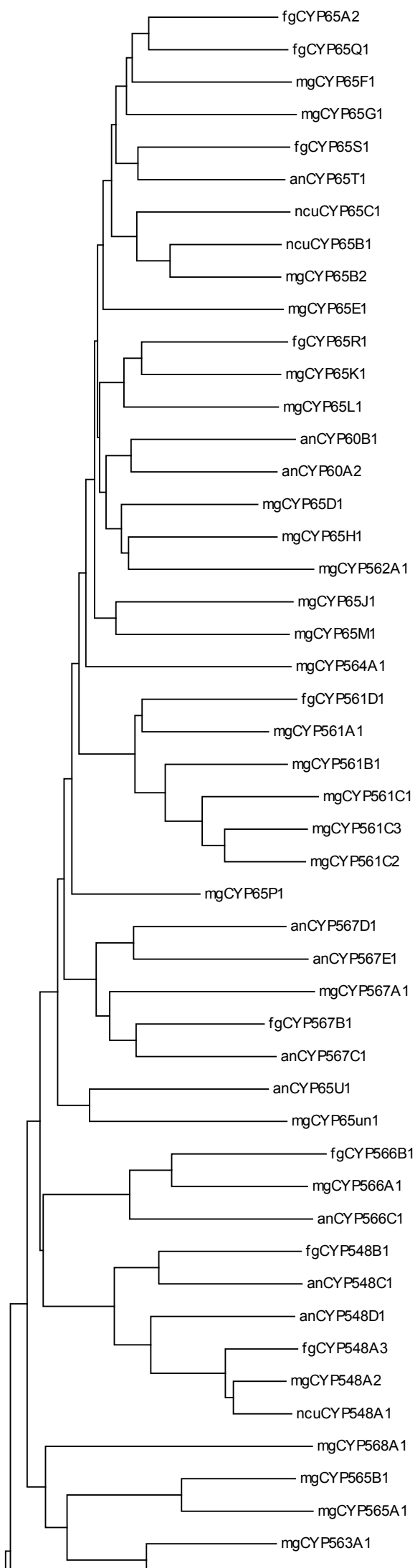

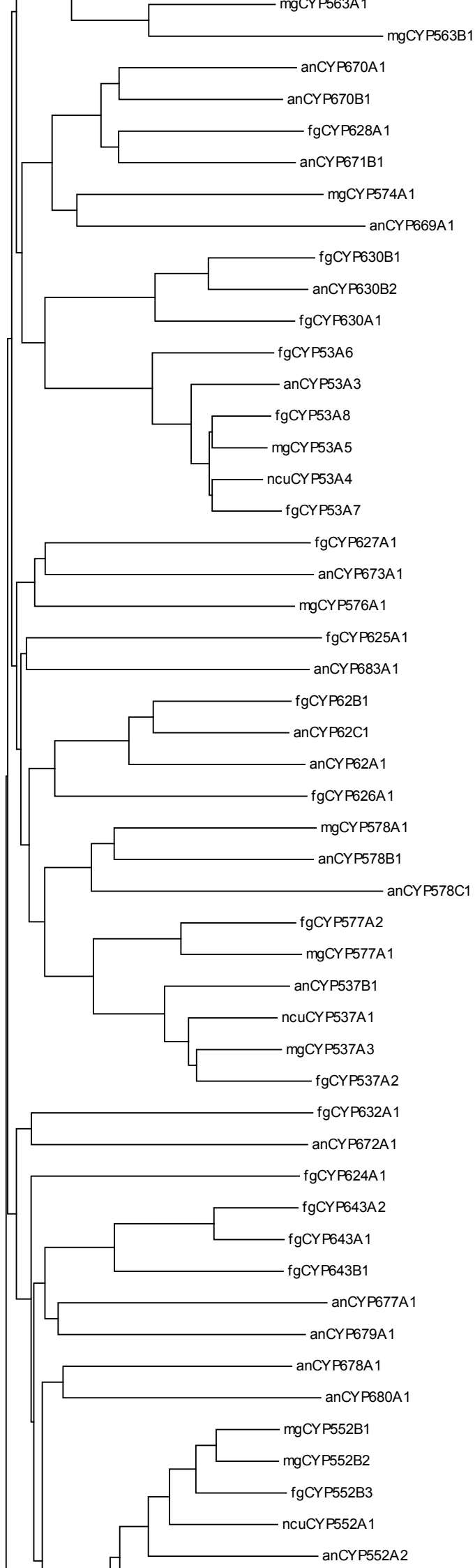

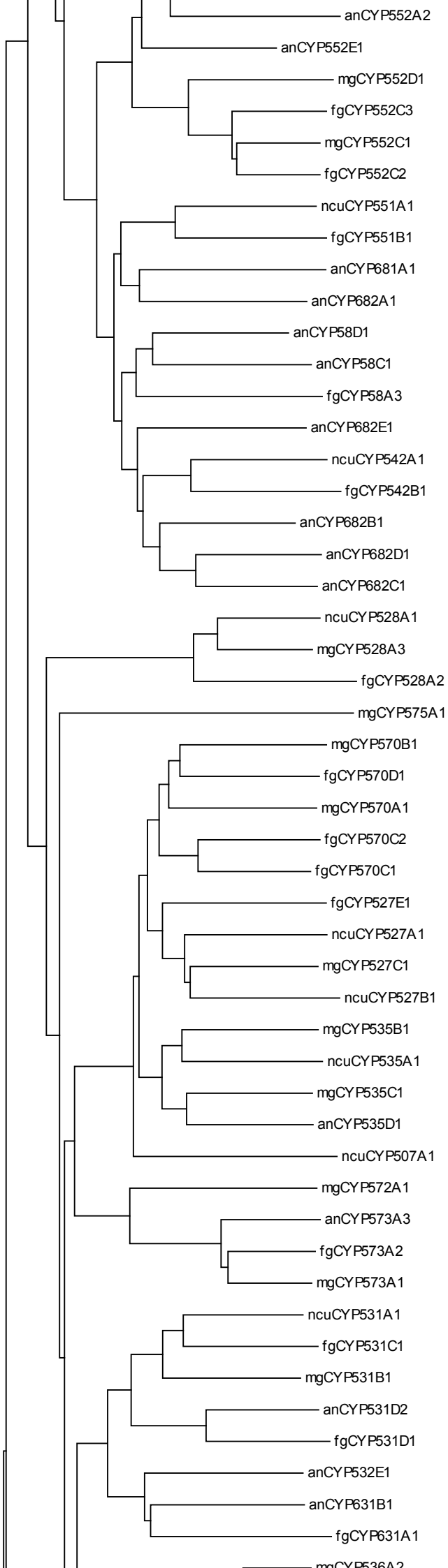

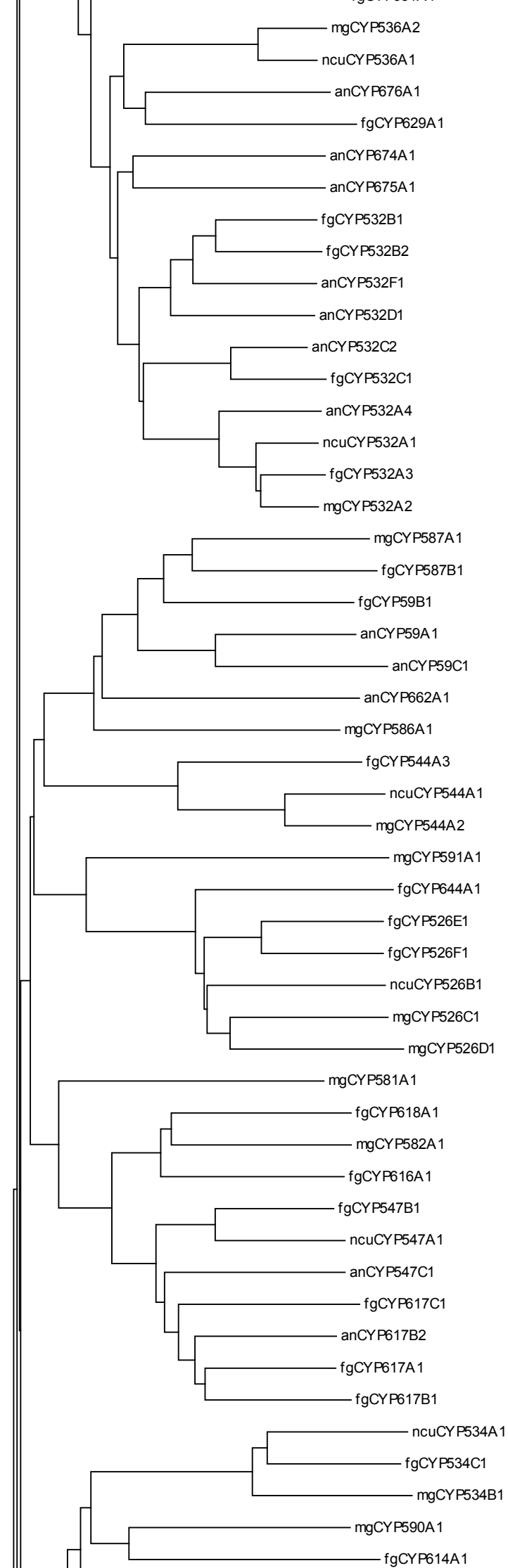

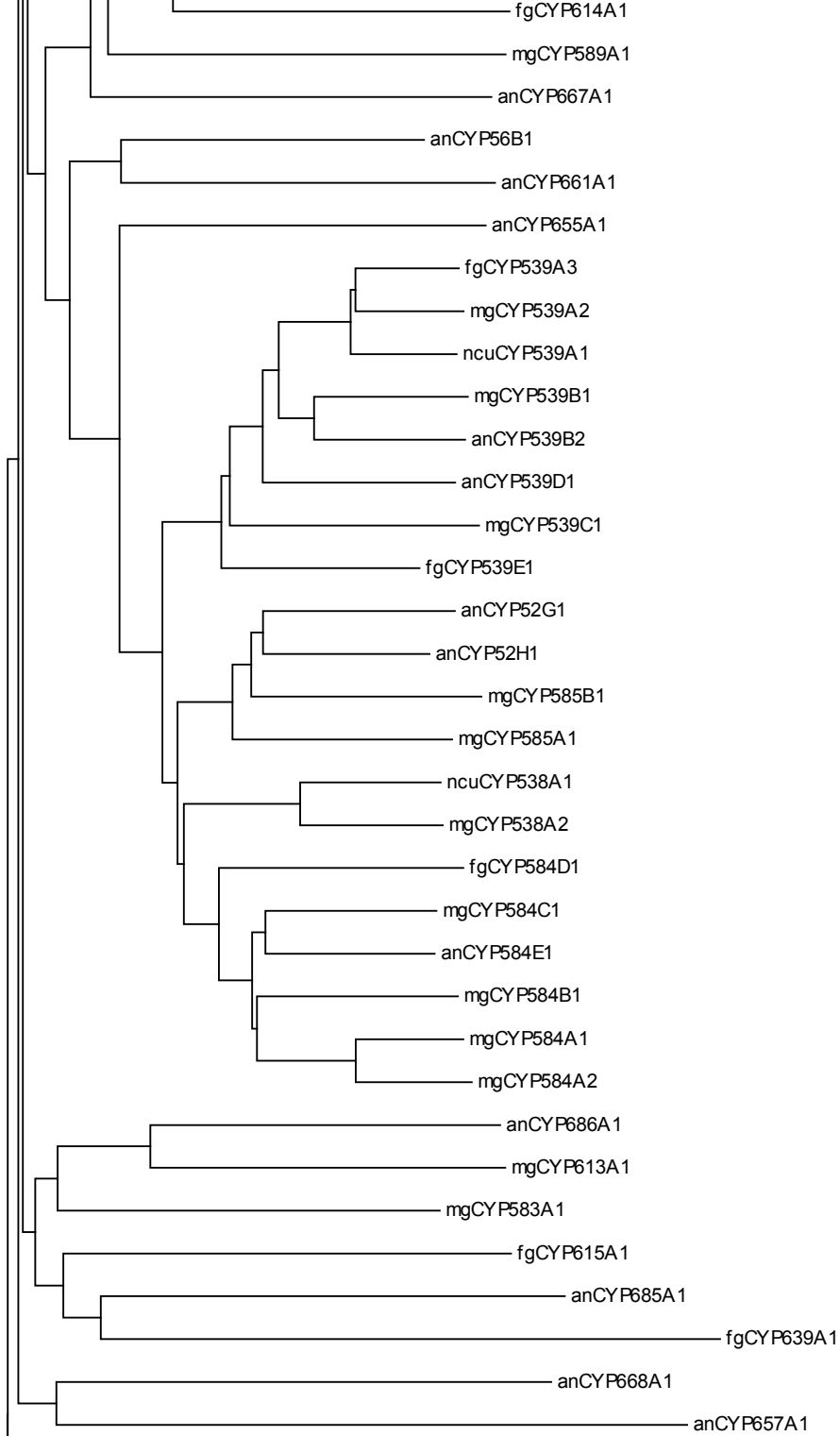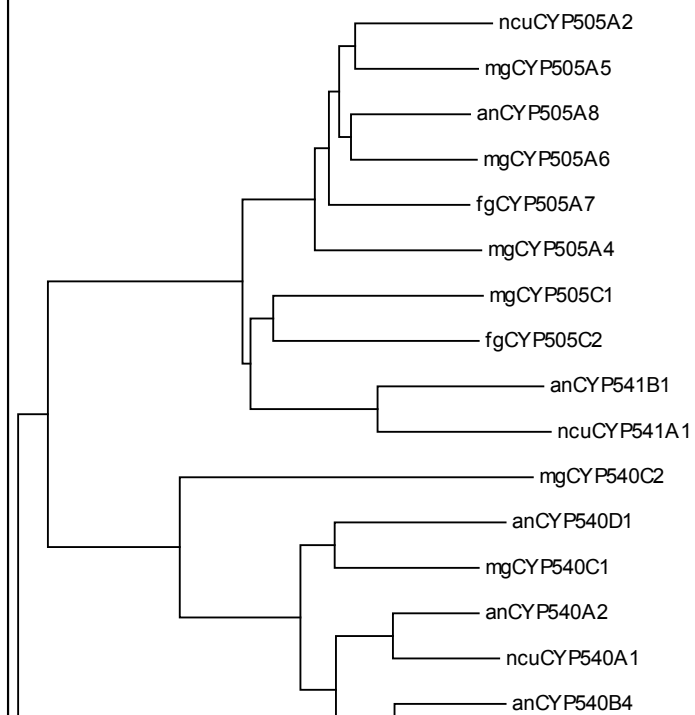

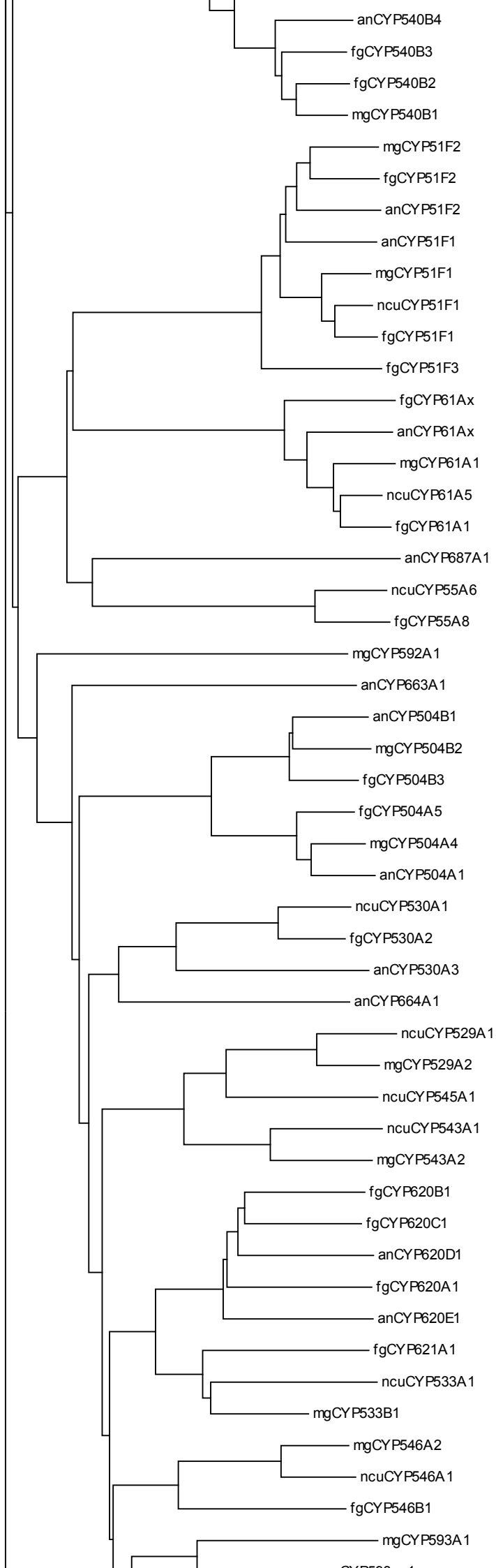

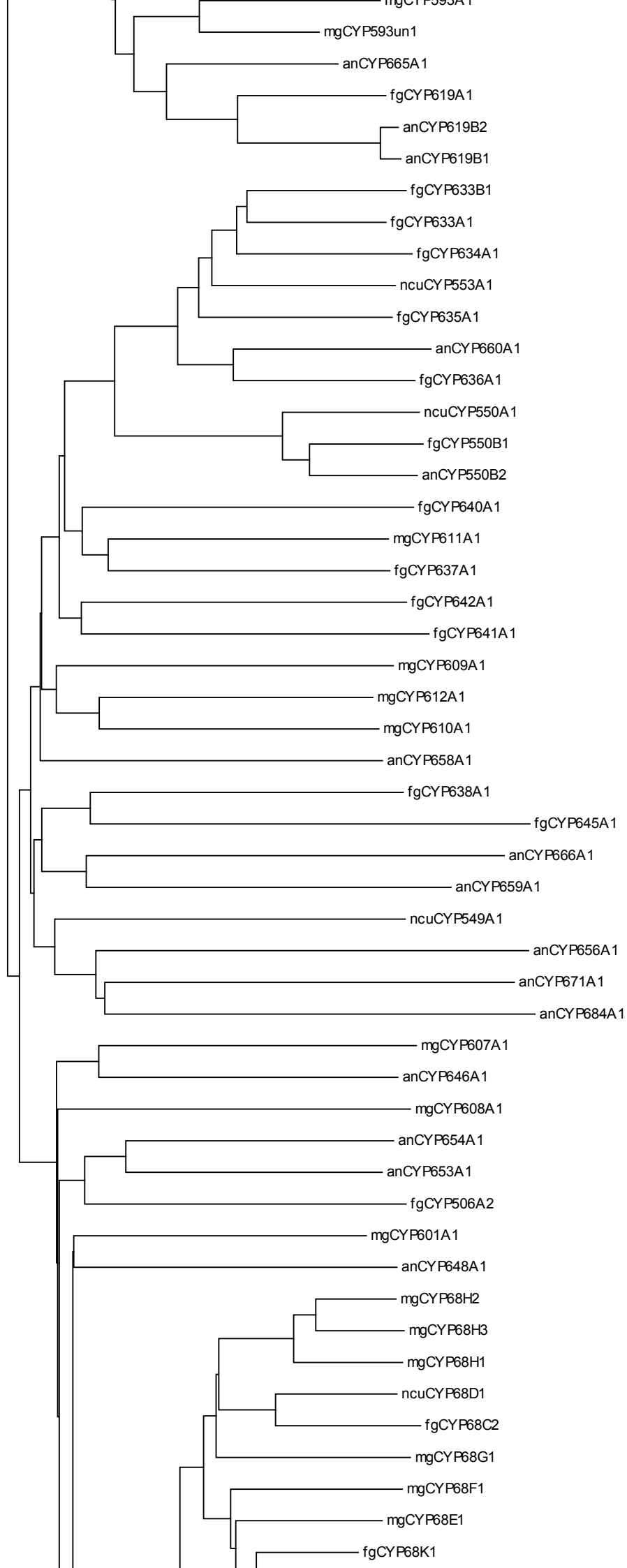

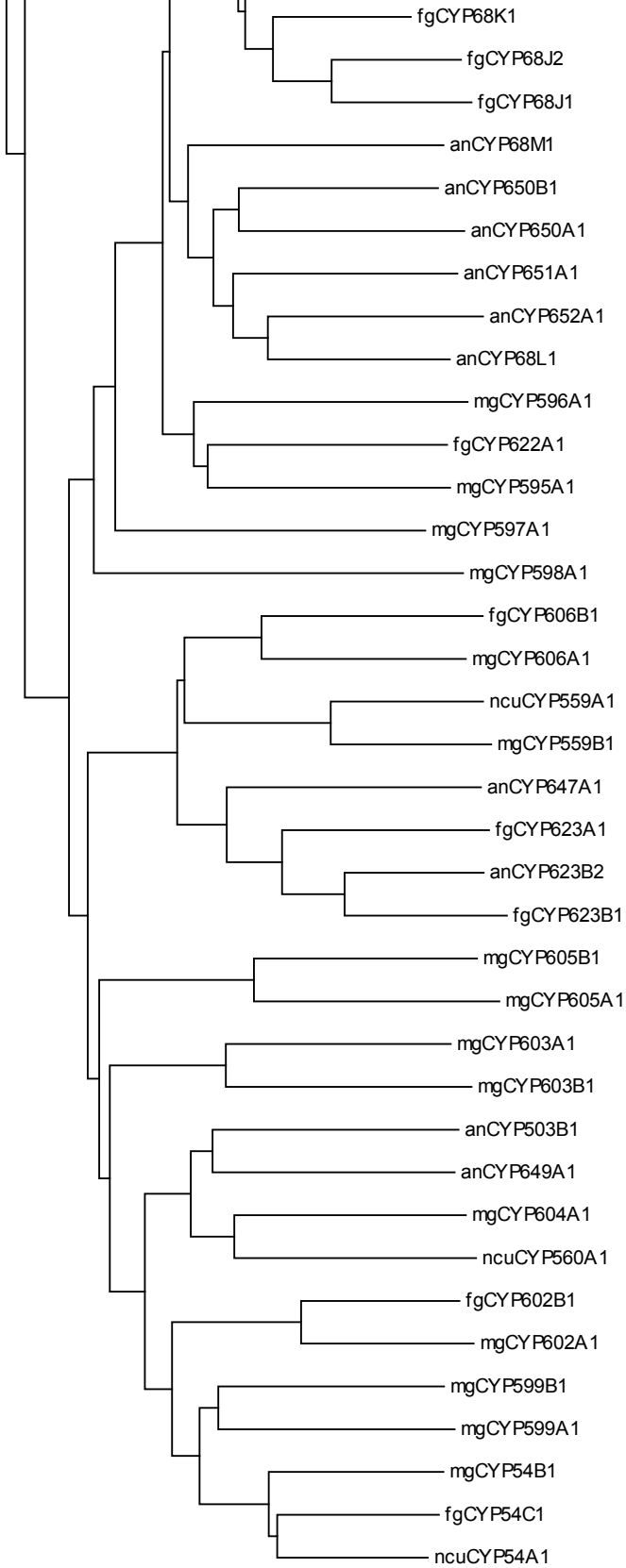

0.5

Supplement: Additional file 2 — NJ tree of 376 P450s from 4 filamentous Ascomycetes [file 1471-2148-7-30-S2.pdf]

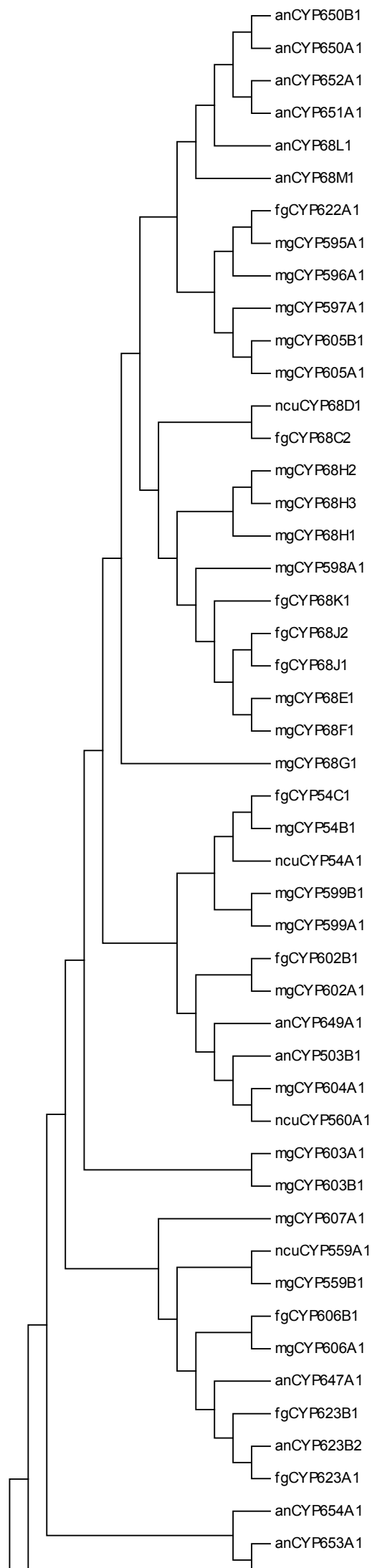

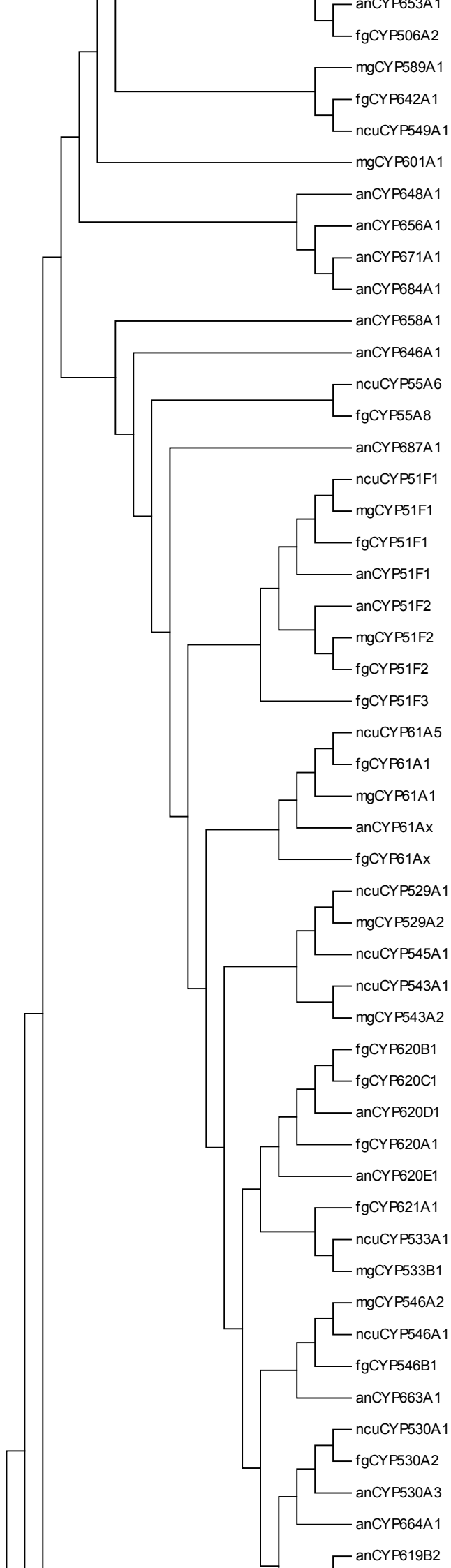

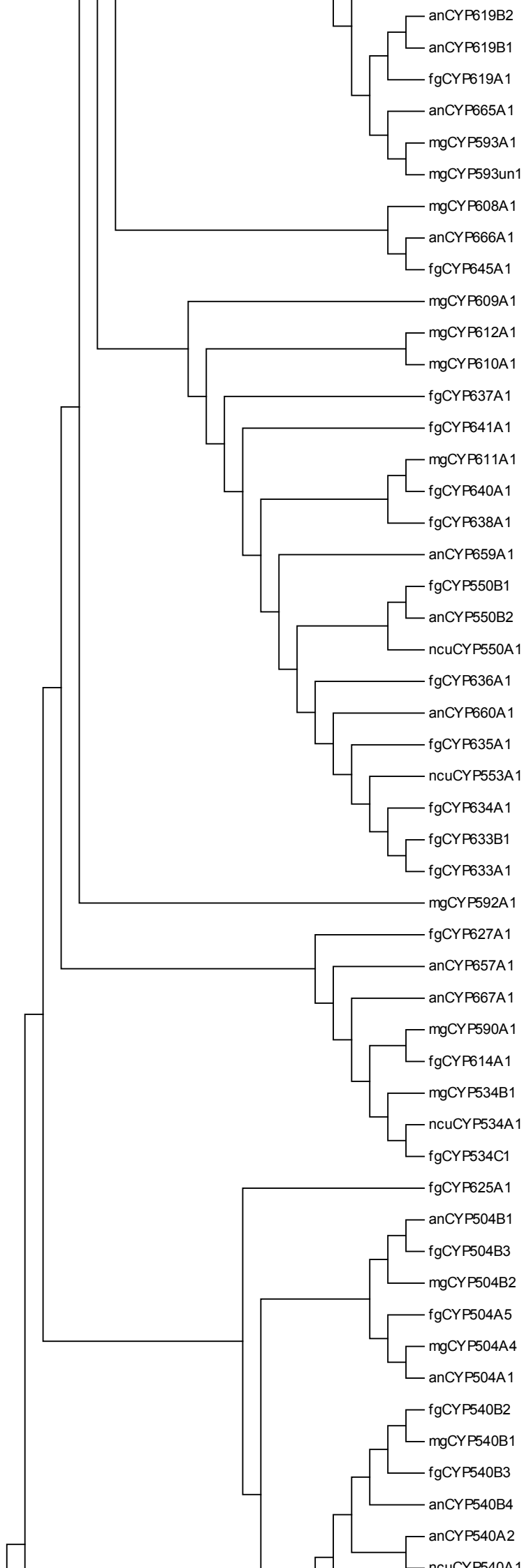

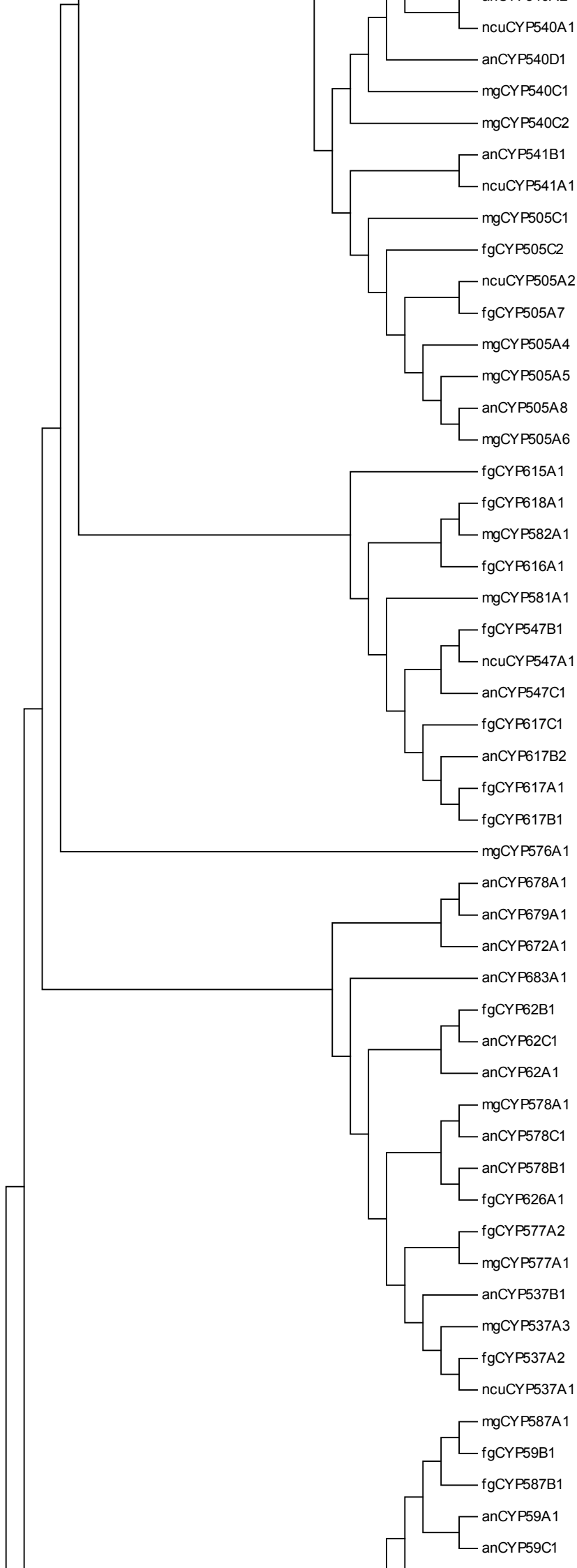

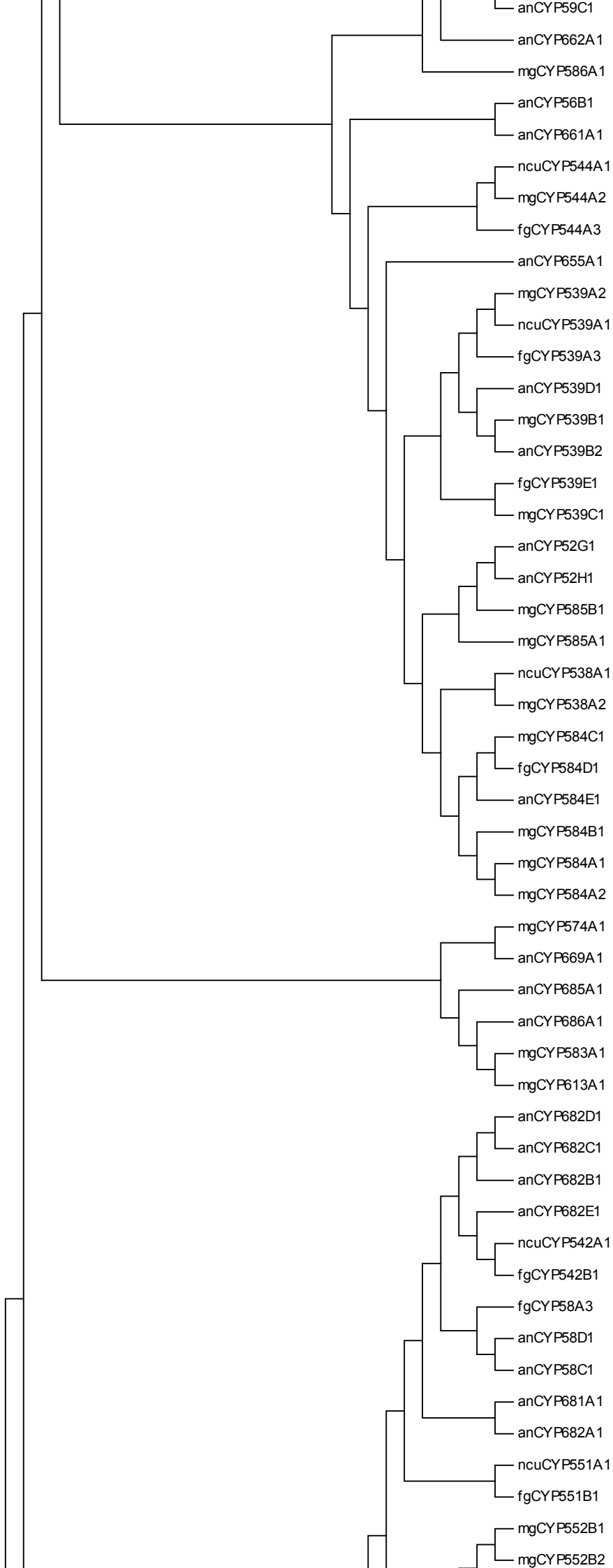

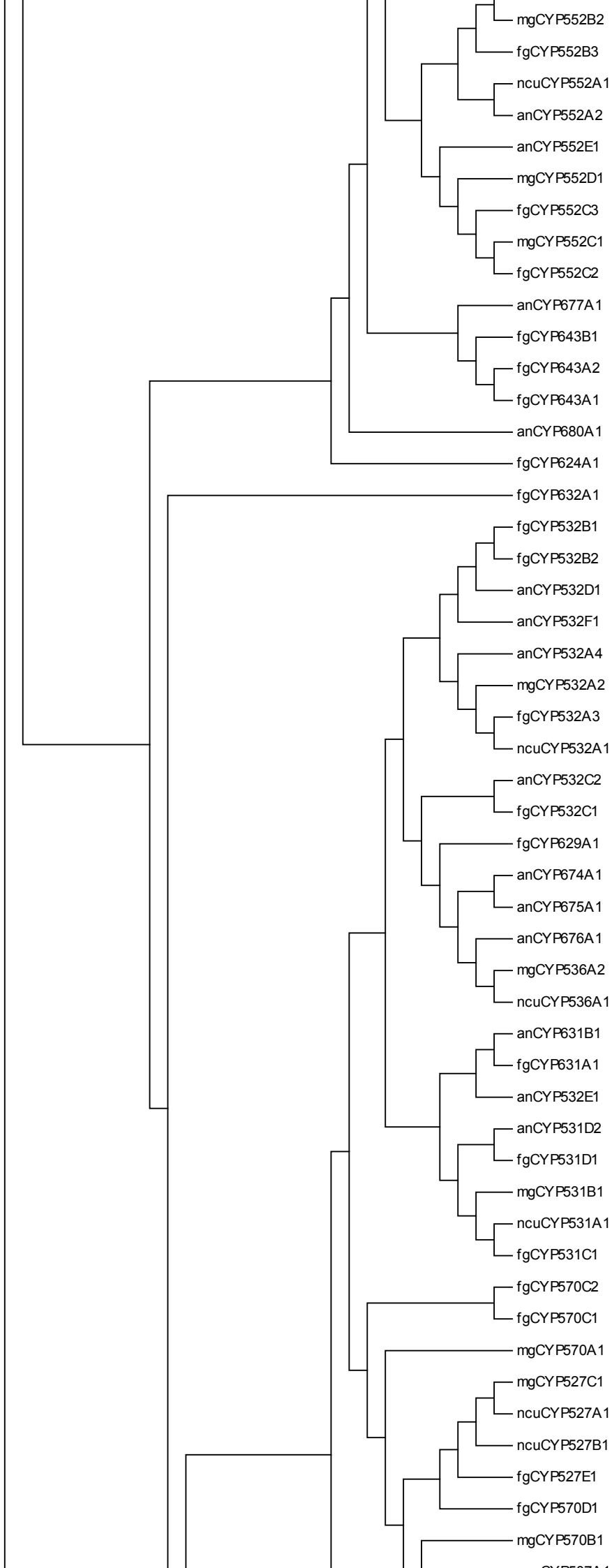

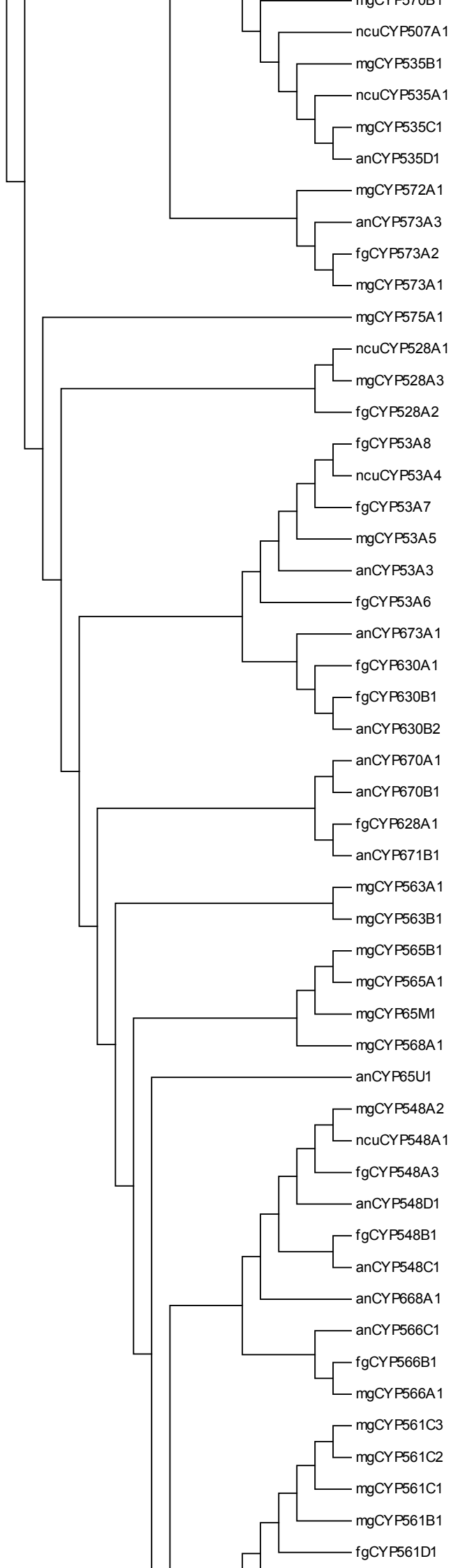

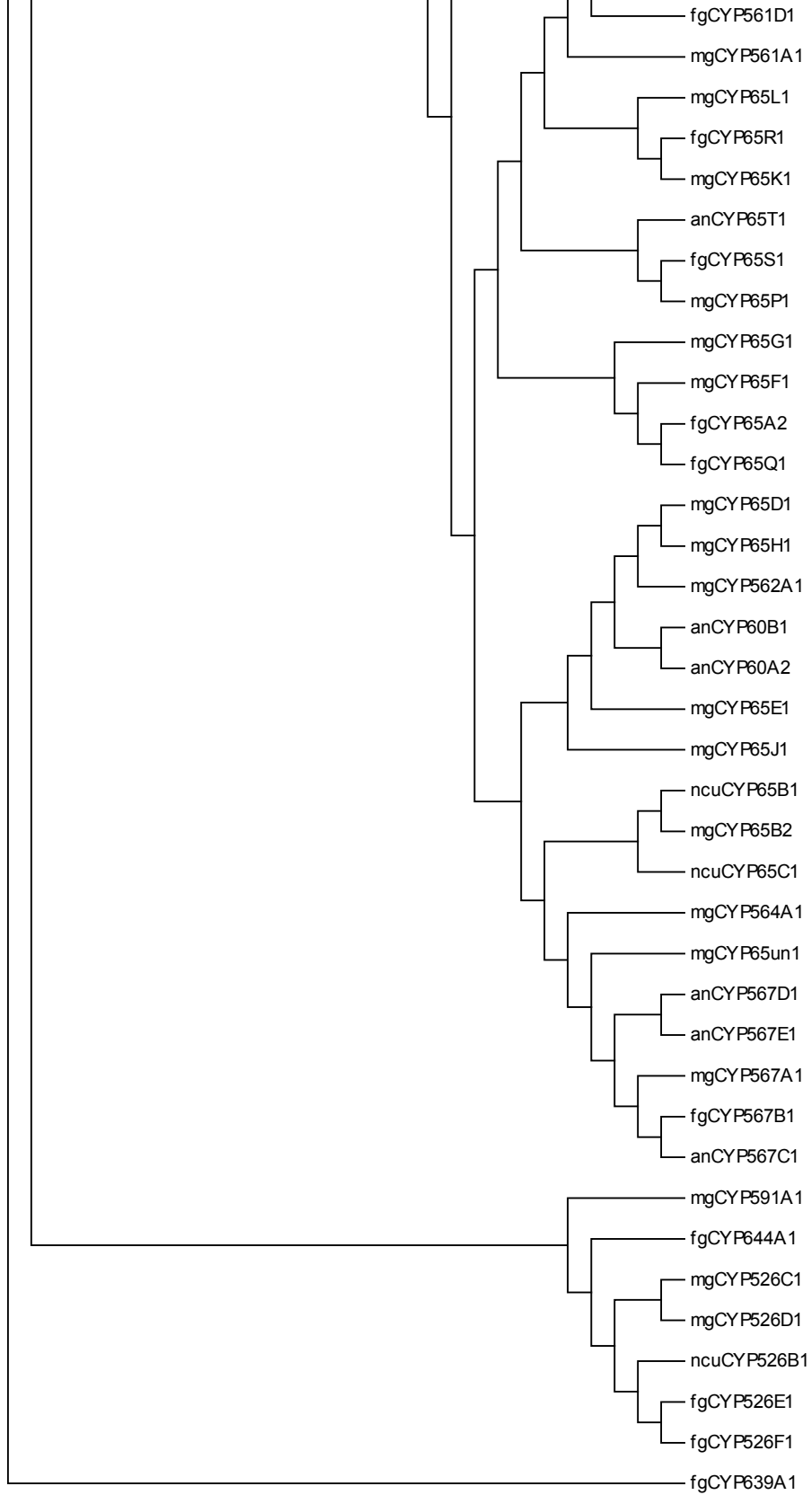

Supplement: Additional file 3 — MP tree of 376 P450s from 4 filamentous Ascomycetes [file 1471-2148-7-30-S3.pdf]

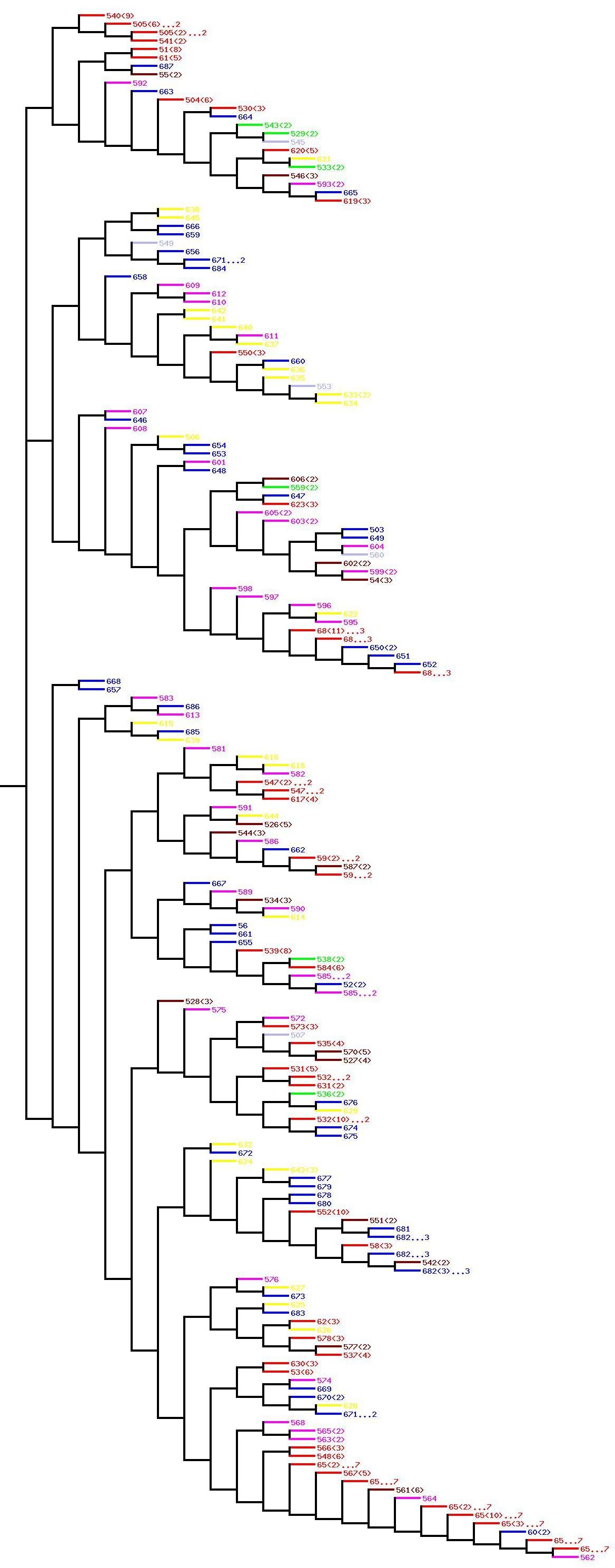

Supplement: Additional file 4 — Collapsed NJ tree of 376 P450s from 4 filamentous Ascomycetes. Each collapsed branch is followed by a number representing the CYP family taxon name. The number in <> after the CYP family name represents the number of genes under this collapsed branch. Number after the ...represents the total number of branches containing other members of the same CYP family in this collapsed phylogeny. [file 1471-2148-7-30-S4.jpeg]

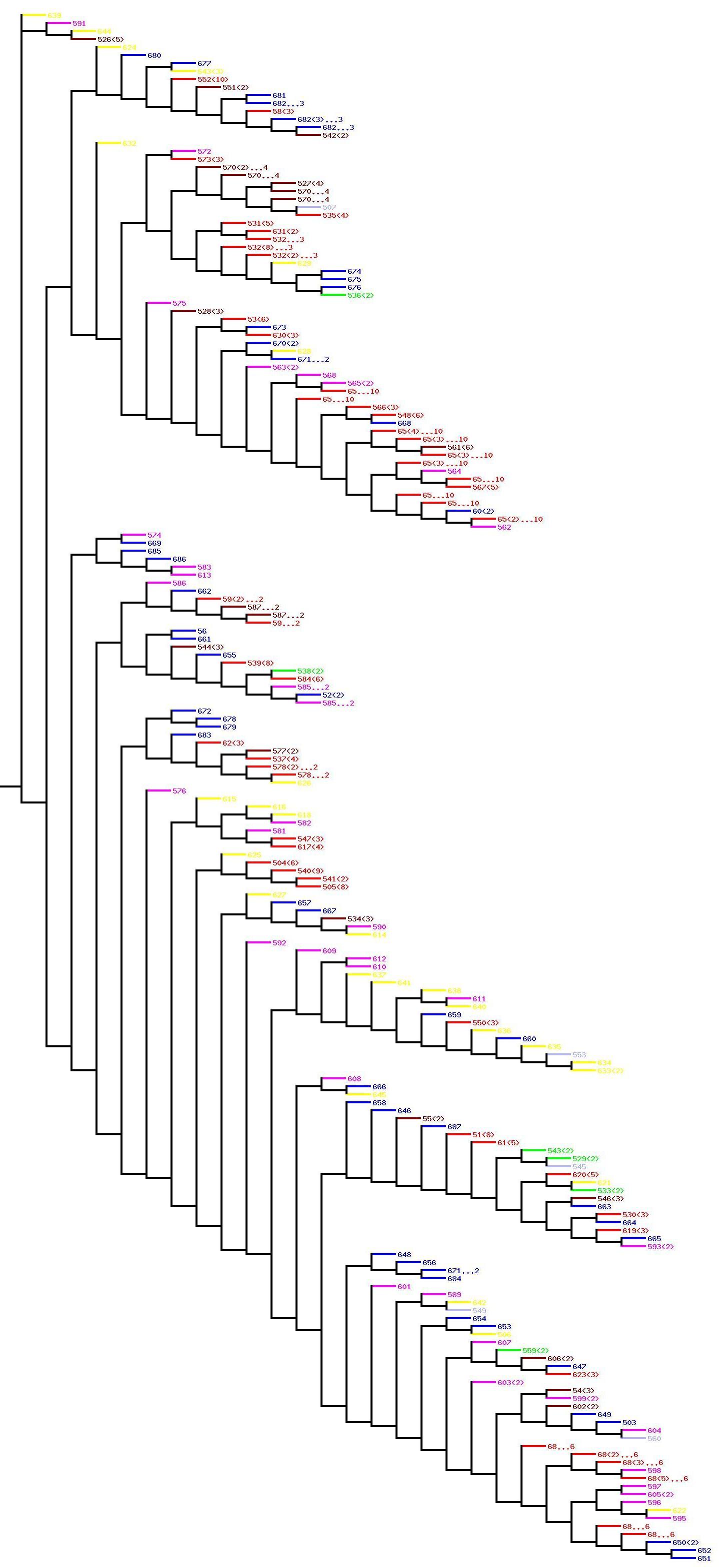

Supplement: Additional file 5 — Collapsed MP tree of 376 P450s from 4 filamentous Ascomycetes. Each collapsed branch is followed by a number representing the CYP family taxon name. The number in <> after the CYP family name represents the number of genes under this collapsed branch. Number after the ...represents the total number of branches containing other members of the same CYP family in this collapsed phylogeny. [file 1471-2148-7-30-S5.jpeg]

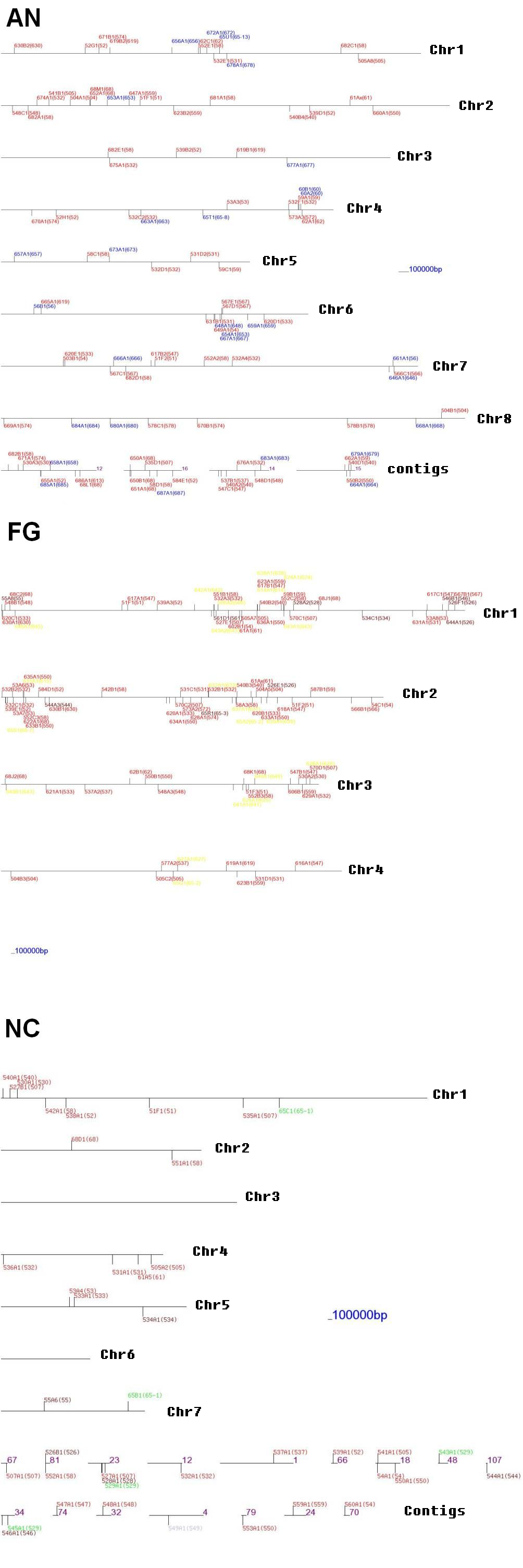

Supplement: Additional file 9 — The physical location of all P450 genes in AN, FG and NC genomes. Each horizontal line represents a chromosome. Those at the bottom followed by a number represent unanchored contigs. A vertical bar above (on 5-3' strand) or below (3'-5' strand) horizontal line marks the position and orientation of each CYP gene, the name of which is marked by omitting the leading "CYP". The number after each gene in parenthesis represents the clan to which this gene was assigned. For colour codes, see Figure 2. [file 1471-2148-7-30-S9.tiff]

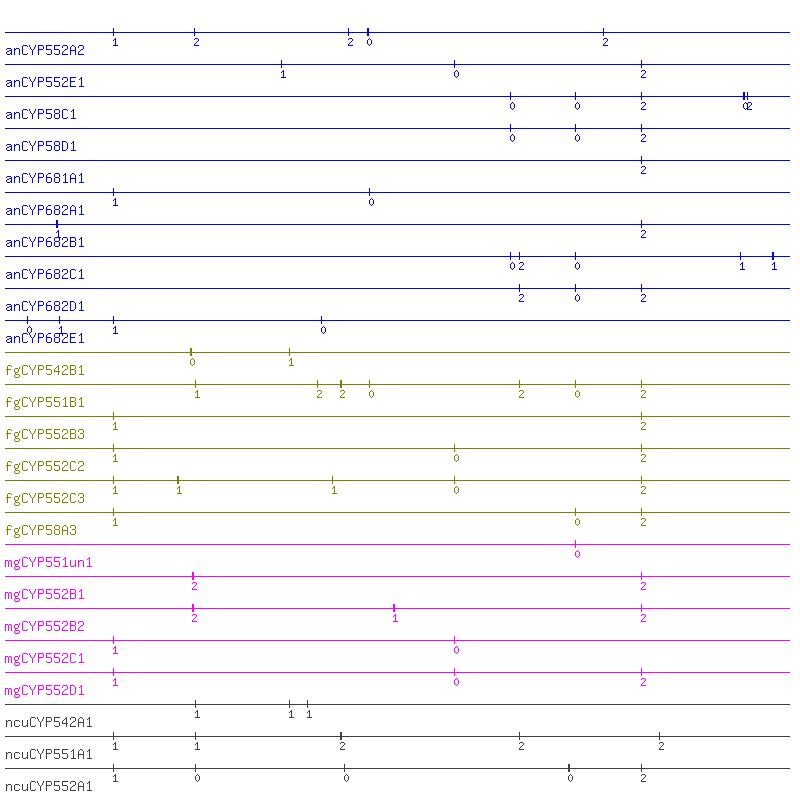

Supplement: Additional file 13 — Intron-exon organization of clan 58. Horizontal lines represent P450 amino acid sequences. A vertical bar on the horizontal line represents an intron. The number under the bar represents the intron phase. [file 1471-2148-7-30-S13.tiff]
